# Phenolic Profile, Antioxidant Activity, Chemical Composition, and Elements of Merlot Wine Stored in Toasted Oak Barrels

**DOI:** 10.3390/foods13244100

**Published:** 2024-12-18

**Authors:** Anita Pichler, Ivana Ivić, Jurislav Babić, Josip Mesić, Ina Ćorković, Tanja Marković, Mirela Kopjar

**Affiliations:** 1 Faculty of Food Technology Osijek, Josip Juraj Strossmayer University of Osijek, F. Kuhača 18, 31000 Osijek, Croatia; anita.pichler@ptfos.hr (A.P.); jurislav.babic@ptfos.hr (J.B.); ina.corkovic@ptfos.hr (I.Ć.); mirela.kopjar@ptfos.hr (M.K.); 2 Faculty of Tourism and Rural Development, Josip Juraj Strossmayer University of Osijek, Vukovarska 17, 34000 Požega, Croatia; jmesic@ftrr.hr; 3 Teaching Institute of Public Health for the Osijek-Baranja County, Franje Krežme 1, 31000 Osijek, Croatia; tanja.markovic.zzjz@gmail.com

**Keywords:** Merlot red wine, oak barrel, toasting, phenolic profile

## Abstract

Wine ageing represents an important stage during wine production when the final wine composition is formed. In this study, 2020 and 2021 vintage Merlot red wines were subjected to 12-month ageing in a stainless-steel tank, Excellence oak barrels with medium, medium-plus and medium-long toasting, and a Premium oak barrel with medium toasting. The aim was to investigate the influence of different ageing vessels on the main chemical composition, element content, phenolic profile, antioxidant activity, and wine colour during ageing. The results showed that changes in ethanol, total sugars, pH, and density were minimal, mostly not significant. Slight changes in malic and lactic acid concentration occurred due to malolactic fermentation. Statistically, more changes that are significant occurred in the phenolic profile, and they affected the antioxidant activity of the wine. In both wine vintages, anthocyanin content decreased, followed by an increase in polymeric colour. Elements and individual phenolic compounds changed significantly, depending on vessel type, ageing time, wine vintage, and initial concentrations. The PCA biplots of the mentioned compounds showed that vessel type had a significant influence on wine composition, especially after 12 months of ageing. According to the CIELab parameters, a slight colour change occurred in both wine vintages, but this is not visible to the human eye. According to the obtained results, various changes in the phenolic profile of Merlot wine occurred during ageing, which strongly depended on the ageing vessel used, the ageing time, and the initial wine composition.

## 1. Introduction

Merlot red grape variety was first cultivated in Bordeaux, France. Today, this grape variety and Merlot red wine are widespread throughout the world due to the grape variety’s resistance to colder climates and the possibility to produce a full-bodied wine of dark red to purple colour, with high phenolic content and velvety tannins. Depending on the production method and consumers’ requirements, it is suitable for fast maturation and short ageing, but it can also mature and age for decades, developing special flavour, scent, colour, and chemical composition [1,2]. The chemical composition of wine consists of many different chemical compounds: water, ethanol, sugars, acids, higher alcohols, terpenes, esters, phenolic compounds, trace elements, etc. [3]. The red wine composition during ageing depends on several factors, including wine variety, initial chemical and phenolic composition, ageing vessel type (material, volume, etc.), the presence of oxygen, ageing time, and others [4,5]. The initial chemical and phenolic composition depends first on the grape variety, and environmental conditions in the vineyard (temperature, sunshine hours, precipitation, soil composition, etc.), and then vineyard practices and vinification techniques [6]. Rouxinol et al. [7] observed that the synthesis of certain phenolic compounds could be affected by both ultraviolet radiation and drought stress, although grape variety was found to have a greater impact on the total phenolic content compared to climatic factors. Furthermore, various vinification techniques, such as those applied during pressing, maceration, filtration, fermentation, clarification, maturation, and ageing, significantly influence the chemical and phenolic profiles of the resulting wine [8,9].

Phenolic compounds in wine encompass a diverse range of substances, generally categorised into flavonoids and non-flavonoids. Within grape berries, these compounds are primarily found in the skins and seeds, and their incorporation into must or wine predominantly occurs during processes such as crushing, maceration, and fermentation. Red wines typically contain between 1800 and 3000 mg/L of total phenolic compounds [10,11]. Among these, tannins are recognised as a significant subgroup of phenolic compounds. They play a critical role in shaping the astringency and bitterness of wine, stabilising its colour, and exhibiting antioxidant properties [12]. There are two main sources of wine tannins: grape tannins (condensed tannins or proanthocyanidins) and tannins derived from wooden barrels used for wine ageing (for example, gallotannins) [12,13].

Further, many elements are part of wine composition and, like the ones analysed in this study (boron, sodium, aluminium, calcium, vanadium, chromium, manganese, iron, cobalt, nickel, copper, zinc, arsenic, selenium, strontium, molybdenum, tin, antimony, barium, and led), are mostly naturally present in wine in low concentrations. They contribute to the wine taste, and some of them have a beneficial influence on human health, like Ca, Cu, Zn, etc. On the other side, a negative influence on wine composition or even a possible toxic effect could be a result of an excessive concentration of some elements, for example, Pb [14,15].

The above-mentioned phenolic compounds content, elements, and chemical composition of wine depend on a range of factors, starting from vineyard conditions and viticultural and vinification techniques, including ageing and maturation. Ageing and maturation during the wine production process represent a crucial stage when the final chemical composition and colour of wine are formatted. Wine ageing differs for each wine type, and the final product depends on conditions during this stage (time, temperature, vessel type, etc.) [16]. It could be conducted in different vessels: stainless-steel tanks and wooden barrels are most often used. Stainless steel is a durable material that is chemically inert, easy to maintain, and suitable for wine storage. It usually preserves original wine composition, without adding or taking anything from it [17]. Unlike stainless steel, wooden barrels react with the wine, and their influence on wine is very complex because it depends on various factors. Main factors include the wood type, age, size, and volume of a barrel, toasting level, and others. One of the main characteristics of wooden barrels is their porosity, which enables the micro-oxygenation of wine [18].

The interaction of the wood surface and wine results in changes in the chemical composition of wine. Raw wood is rich in tannins that can be transferred into wine, and it can even have a negative effect on it. Therefore, wooden barrels are usually toasted before usage. Toasting represents ignition of the inside of a barrel under controlled time and temperature, from 180 to 230 °C, 5 to 10 min, depending on the method (light, medium, or heavy toasting), eliminating wood tannins, forming new compounds that enhance wine aroma with vanilla and smoke aromas, soften wine tannins, stabilise wine colour pigments, and influence the wine chemical composition [19,20]. Depending on the required wine characteristics, flavour, and scent, different toasting levels could be applied.

In a previous study, Chira and Teissedre (2015) [21] used barrels made of different oak species (*Q. robur*, *Q. petraea*, and *Q. alba*) from different forests with light, noisette, medium toast, medium toast with watering, medium toast with toasted head, medium-plus toast, and medium-plus toast with watering for Merlot wine storage through 12 months. They concluded that each oak type and origin significantly influenced ellagitannins, whiskey lactones, eugenol, wine astringency, and woody aroma in Merlot wines. Further, the toasting methods had a significant influence on all studied variables of Merlot phenolic and aroma profile, including wine flavour and taste. In another study conducted by Stavridou et al. [2], the phenolic profiles of Cabernet Sauvignon, Merlot, and Syrah, stored for 4, 6, 7, and 10 months in medium and heavy-toasted French and American oak, were investigated. Regardless of the initial differences among wine varieties, the toasting method of the barrel had a significant, but different, influence on the phenolic profile of the analysed wines. The influence of medium toasting variations on Cabernet Sauvignon composition from three different countries and cellars (France, Italy, and the USA) was also investigated by González-Centeno et al. [22]. Their results showed that, despite using the same wood and the same toasting methods, the interactions between the wine matrix and wooden barrel greatly depend on the initial wine composition. Further, del Fresno et al. [23] also studied the influence of different oak barrels on red wine phenolic and aromatic profiles, and they concluded that the correct choice of barrel type is very important for high-quality wine with desirable composition. In 2021, Pfahl et al. [24] studied red wine maturation in medium oak barrels from different cooperages. They concluded that each barrel-to-barrel variation influenced the chemical and phenolic composition of the red wine and that more research is required in this field.

Therefore, in this study, for additional investigation, medium, medium-plus, and medium-long toasting of oak barrels with two-grain density were used for the ageing of two vintages of the Merlot red wine variety, and they were compared to the ageing in a stainless-steel tank. The influence of the mentioned vessels on wine chemical composition, element content, phenolic compounds content, antioxidant activity, and colour were analysed during 12 months of ageing. This study investigated two Merlot vintages to understand the changes or similarities in chemical and phenolic composition that winemakers can expect each year using identical vinification procedures and ageing vessels, including stainless-steel tanks and toasted barrels. A hypothesis can be made: no significant changes in the ageing vessel influence on main chemical composition could be observed, but the differences in phenolic composition and trace elements could be greatly influenced by different initial wine composition, which is a result of different vintage climatic conditions.

## 2. Materials and Methods

### 2.1. Reagents and Standards

For the purpose of this study, following reagents and standards were necessary: elements standards: Be, Cr, Mn, Co, Ni, Cu, As, Mo, Ag, Cd, Pb, Na, Al, Ca, Fe, Zn, Sr, and Ba (Inorganic Ventures, Christiansburg, VA, USA); ultrapure nitric acid (65%) (Fisher Scientific, Hampton, NH, USA); Trolox, 2,2-diphenyl-1-picrylhydrazil (DPPH), 2,2-azinobis(3-ethylbenzothiazoline sulfonic acid) (ABTS), 2,4,6-tripyridyl-s-triazine (TPTZ), 4-(dimethylamino)cinnamaldehyde (DMAC), rhodanine, potassium persulfate, gallic acid monohydrate, aluminium chloride, and quercetin dihydrate (Sigma-Aldrich, St. Louis, MO, USA); HPLC standards (gallic, caffeic, caftaric, coutaric and p-coumaric acid, quercetin, hyperoside, (+)-catechin, and (−)-epicatechin) (Sigma-Aldrich Chemie Gmbh, Steinheim, Germany) and malvidin-3-glucoside and dephinidin-3-glucoside (Extrasynthese, Genay, France); Folin–Ciocalteu reagent, sodium nitrite, sodium hydroxide, sodium carbonate, sodium acetate, potassium chloride, potassium bisulphite, potassium hydroxide, and sulphuric and hydrochloric acid (Kemika, Zagreb, Croatia); ammonium acetate, ferric chloride hexahydrate, and sodium acetate trihydrate (Gram-Mol, Zagreb, Croatia); HPLC-grade methanol and neocuproine (Merck, Darmstadt, Germany); HPLC-grade phosphoric acid (Fluka, Buchs, Switzerland).

### 2.2. Oak Barrels

For this study, oak barrels crafted by the Auric Barrels cooperage (Našice, Croatia) were utilised. These barrels are made from a blend of sessile oak (*Quercus petraea* L.) and pedunculated oak (*Quercus robur* L.) in a 70:30 ratio, sourced from trees aged 120 to 140 years. The cooperage produces two types of barrels: Excellence barrels, characterised by 3–5 grains per centimetre, and Premium barrels, with 5–7 grains per centimetre. The staves for both barrel types undergo air-drying for 24 to 36 months. In this research, three Excellence barrels per wine vintage were used, featuring medium toasting (60 min, temperatures increasing from 100 to 190 °C), medium-plus toasting (60 min, from 110 to 205 °C), and medium-long toasting (65 min, from 120 to 210 °C). Additionally, one Premium barrel with medium toasting was included.

### 2.3. Merlot Wine

Merlot grapes used in this study were cultivated in the Kutjevo vineyard, with the harvest date determined based on grape ripeness. In 2020, the harvest occurred on November 11, while for the 2021 vintage, it took place on November 1. The grapes were crushed, and the resulting mash macerated in a stainless-steel Vinimatic for 12 days, with twice-daily punch-downs. Approximately 3600 L of grapes was macerated in total. Following maceration, the mash was pressed, and the resulting must was transferred to a stainless-steel tank for fermentation. Fermentation was carried out using *Saccharomyces* Siha Finesse Red yeasts (BHF Technologies, Oakleigh, Australia) at temperatures ranging between 23 and 25 °C. The resulting wine was completed in early March 2020 (referred to as the initial Me20 sample) and May 2021 (initial Me21 sample). Malolactic fermentation was not induced through inoculation. Sulphur dioxide (SO_2_) was first added after grape crushing, then again post fermentation and after the lees were removed, maintaining a free SO_2_ concentration of 20 mg/L. After the initial sampling, the wines were divided across five different ageing vessels: a stainless-steel tank (SST), Excellence oak barrels with medium toasting (EMT), medium-plus toasting (EMT+), and medium-long toasting (EMLT), as well as a Premium oak barrel with medium toasting (PMT). All vessels were stored under identical conditions in a cellar maintained at 16–18 °C. Sampling was conducted every three months over 12 months of ageing, with two 750 mL samples taken from each vessel. The same protocol was followed for both vintages (2020 and 2021), with the only distinction being the climatic conditions in the vineyard that affected the harvest timing. Detailed data on air temperature, sunshine duration, and precipitation during the two vintages were provided in a previous study [5].

### 2.4. Main Chemical Composition

OenoFoss^TM^ (Foss, Hilleroed, Denmark) was used for chemical composition analysis. It was calibrated with ready-to-use standard solutions (calibration package from Foss, Hilleroed, Denmark) for must and fermented wine and with the FTIR Calibrator software 2.0 (Foss, Hilleroed, Denmark) for determination of ethanol, total sugars, pH, total and volatile acidity, malic acid, and lactic acid. Calibration ranges were as follows: ethanol (0–19 vol.%), total sugars (glucose/fructose, 0–20 g/L), pH (2.6–4.0), total acidity (0–11.7 g/L, expressed as tartaric acid), volatile acidity (0–1.4 g/L, expressed as acetic acid), lactic acid (0–4.6 g/L), and malic acid (0–7.0 g/L).

### 2.5. Phenolic Composition and Antioxidant Activity

The total polyphenol and flavonoid content, monomeric anthocyanins, polymeric colour, and antioxidant activity were analysed spectrophotometrically, following the methodologies outlined in detail in our previous study [25]. Polyphenol content was determined using the Folin–Ciocalteu reagent, with results expressed as gallic acid equivalents (g GAE/L). Flavonoids were quantified as catechin equivalents (g CE/L). Monomeric anthocyanins and polymeric colour were measured using the pH-differential method. Antioxidant activity was assessed using the DPPH, ABTS, FRAP, and CUPRAC assays, with results standardised to Trolox equivalents (μmol/100 mL). For the DPPH assay, 0.2 mL of the sample was mixed with a DPPH solution prepared in 96% ethanol, and the absorbance was recorded at 517 nm after 15 min. The ABTS assay involved preparing an ABTS solution by reacting 7 mM ABTS with 2.45 mM potassium persulfate (1:1). This solution was diluted with 96% ethanol (2:70 ratio) before analysis, and 0.2 mL of the sample was combined with 3.2 mL of the diluted ABTS solution, with absorbance measured at 734 nm after 95 min. The FRAP assay was performed by mixing 0.2 mL of the sample with 3 mL of FRAP reagent, and absorbance was measured at 593 nm after 15 min. The FRAP reagent consisted of 300 mM sodium acetate buffer (pH 3.6), 10 mM TPTZ in 40 mM HCl, and 20 mM FeCl_3_ × H_2_O. The CUPRAC method involved combining 1 mL of 10 mM CuCl_2_ × H_2_O, 1 mL of 7.5 mM neocuproine, and 1 mL of 1.0 M ammonium acetate in a test tube, followed by the addition of 0.2 mL of the sample and 0.9 mL of distilled water (4.1 mL total volume). Absorbance was recorded at 450 nm after 30 min. DMAC assay [26] was used for the quantification of condensed tannins that were expressed as proanthocyanidin B2 equivalents (mg/L). Rhodanine assay according to Inoue and Hagerman [27] was used for the quantification of hydrolysable tannins (gallotannins), expressed as gallic acid equivalents (mg GAE/L). For each assay, water was used as blank.

A high-performance liquid chromatography (HPLC) system from Agilent Technologies (Santa Clara, CA, USA) was utilised to identify and quantify individual phenolic compounds. The system included a Poroshell 120 EC-C18 column (4.6 × 100 mm, 2.7 μm), a quaternary pump, and a diode array detector (DAD). The operating conditions were based on the protocol described by Ivić et al. [28]. In summary, the mobile phases consisted of 0.1% H_3_PO_4_ (Phase A) and 100% methanol (Phase B). Two separate methods were employed. For anthocyanins, a 20 μL injection volume was used with a gradient program as follows: 0–38 min, 3–65% B; 38–45 min, 65% B. For other phenolic compounds, the injection volume was 10 μL, and the gradient profile was as follows: 0 min, 5% B; 3 min, 30% B; 15 min, 35% B; 22 min, 37% B; 30 min, 41% B; 32 min, 45% B; 40 min, 49% B; 45 min, 80% B; 48 min, 80% B; 50 min, 5% B; and 53 min, 5% B. The flow rate was set to 1 mL/min, and the detection wavelength range spanned from 190 to 600 nm.

### 2.6. CIELab Parameters

Colour parameters of samples were determined with the chromometer CR-400 (Konica Minolta, Inc., Osaka, Japan). The following parameters were measured: lightness (L*), redness/greenness (a*), yellowness/blueness (b*), colour saturation (C*), and hue angle (°h). The total colour change (ΔE*) was calculated according to the equation:

ΔE* = [(ΔL*)^2^ + (Δa*)^2^ + (Δb*)^2^]^1/2^ (1)

### 2.7. Element Composition

Wines were collected in metal-free 50 mL plastic tubes and stored at 4 °C until analysis. Before analysis, wine samples were diluted 1:10 in 1% nitric acid. An Agilent 7900 ICP-MS single quadrupole with Octopole Reaction System (ORS) collision/reaction cell was used for the study. The operating conditions are presented in Table 1. The ICP-MS was calibrated before analysis using an Agilent Technologies (Santa Clara, CA, USA) tuning mix (Li, Y, Ce, Tl, Co). The ISTD solution was mixed with the sample, using a mixing tee before the nebuliser. Agilent ICP-MS MassHunter 4.3 Workstation software (version C.01.03) was used to acquire and analyse the data.

### 2.8. Statistical Analysis

All results were reported as the mean of three replicates ± standard deviation, calculated using MS Excel (Microsoft Office Professional, 2016). Correlation coefficients were also computed using the same software. Principal component analysis (PCA) biplots, analysis of variance (ANOVA), and post hoc Fisher’s least significant difference (LSD) test (*p* < 0.05) were performed using STATISTICA 13.1 (StatSoft Inc., Tulsa, OK, USA).

## 3. Results and Discussion

**Figure 1:**
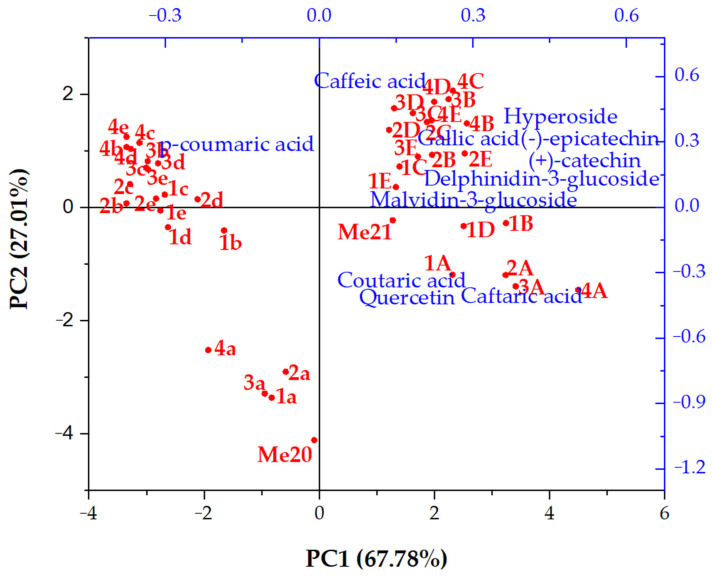
The PCA biplot of individual phenolic compounds identified in 2020 and 2021 Merlot red wines and samples obtained during their 12-month ageing in different vessels. Me20—Merlot vintage 2020 sample before ageing; Me21—Merlot vintage 2021 sample before ageing; a, A—stainless-steel tank; b, B—Excellence barrel with medium toasting; c, C—Excellence barrel with medium-plus toasting; d, D—Excellence barrel with medium-long toasting; e, E—Premium barrel with medium toasting; 1a–1e—sampling in June 2021; 2a–2e—sampling in September 2021; 3a–3e—sampling in December 2021; 4a–4e—sampling in March 2022; 1A–1E—sampling in August 2022; 2A–2E—sampling in November 2022; 3A–3E—sampling in February 2023; 4A–4E—sampling in May 2023.

**Figure 2:**
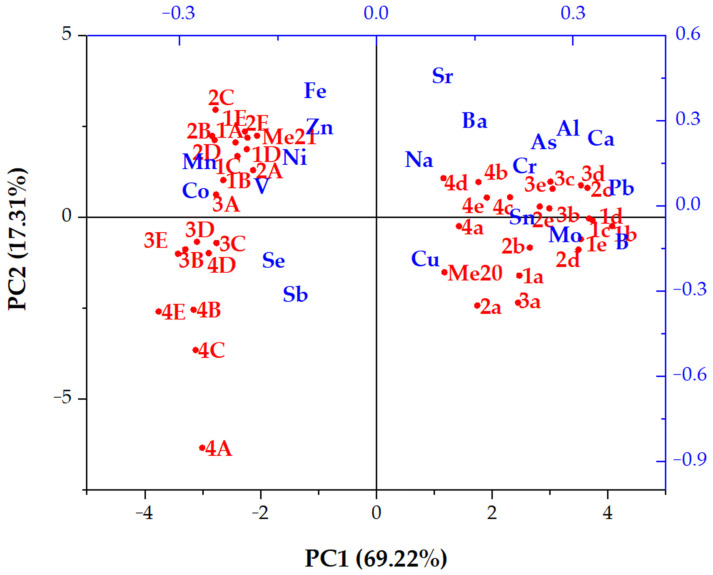
The PCA biplot of elements identified in 2020 and 2021 Merlot red wines and samples obtained during their 12-month ageing in different vessels. Me20—Merlot vintage 2020 sample before ageing; Me21—Merlot vintage 2021 sample before ageing; a, A—stainless-steel tank; b, B—Excellence barrel with medium toasting; c, C—Excellence barrel with medium-plus toasting; d, D—Excellence barrel with medium-long toasting; e, E—Premium barrel with medium toasting; 1a–1e—sampling in June 2021; 2a–2e—sampling in September 2021; 3a–3e—sampling in December 2021; 4a–4e—sampling in March 2022; 1A–1E—sampling in August 2022; 2A–2E—sampling in November 2022; 3A–3E—sampling in February 2023; 4A–4E—sampling in May 2023.

The results of the chemical, phenolic content, and colour analysis of 2020 Merlot and 2021 Merlot red wine and samples obtained during 12-month ageing in SST, EMT, EMT+, EMLT, and PMT are presented in Table 1, Table 2, Table 3, Table 4, Table 5, Table 6, Table 7, Table 8, Table 9, Table 10, Table 11, Table 12 and Table 13 and Figure 1 and Figure 2.

### 3.1. Chemical Composition

Table 2 and Table 3 display the chemical composition of the analysed samples for the 2020 and 2021 Merlot vintages, respectively. 

**Table 2:** Chemical composition of Merlot vintage 2020 and samples obtained during 12-month ageing in different vessels.

Sample	Ethanol (vol.%)	Total Sugar (g/L)	pH	Total Acidity (g/L)	Volatile Acidity (g/L)	Malic Acid (g/L)	Lactic Acid (g/L)	Density (kg/L)
Me20	14.20 ± 0.05 ^ab^	2.10 ± 0.05 ^c^	3.73 ± 0.02 ^a^	4.05 ± 0.05 ^a^	0.44 ± 0.01 ^a^	1.00 ± 0.05 ^b^	0.65 ± 0.05 ^ab^	0.9917 ± 0.0001 ^a^
1a	15.05 ± 0.10 ^e^	1.90 ± 0.05 ^b^	3.74 ± 0.02 ^a^	4.25 ± 0.05 ^b^	0.45 ± 0.01 ^a^	1.00 ± 0.05 ^b^	0.60 ± 0.05 ^ab^	0.9914 ± 0.0002 ^a^
2a	15.20 ± 0.10 ^e^	1.90 ± 0.05 ^b^	3.75 ± 0.04 ^a^	4.25 ± 0.05 ^b^	0.46 ± 0.01 ^ab^	1.05 ± 0.05 ^b^	0.55 ± 0.05 ^a^	0.9915 ± 0.0001 ^a^
3a	15.10 ± 0.10 ^e^	1.75 ± 0.05 ^ab^	3.71 ± 0.03 ^a^	4.25 ± 0.05 ^b^	0.47 ± 0.01 ^b^	0.95 ± 0.05 ^b^	0.80 ± 0.05 ^bc^	0.9916 ± 0.0001 ^a^
4a	15.20 ± 0.05 ^e^	1.85 ± 0.05 ^ab^	3.75 ± 0.02 ^a^	4.20 ± 0.05 ^b^	0.48 ± 0.01 ^b^	0.90 ± 0.05 ^ab^	0.80 ± 0.05 ^bc^	0.9915 ± 0.0001 ^a^
1b	14.15 ± 0.05 ^a^	2.00 ± 0.05 ^bc^	3.73 ± 0.02 ^a^	4.45 ± 0.05 ^c^	0.48 ± 0.01 ^b^	1.00 ± 0.05 ^b^	0.75 ± 0.05 ^bc^	0.9916 ± 0.0001 ^a^
2b	14.45 ± 0.15 ^bc^	1.90 ± 0.05 ^b^	3.72 ± 0.01 ^a^	4.40 ± 0.05 ^bc^	0.56 ± 0.01 ^d^	1.05 ± 0.05 ^b^	0.80 ± 0.05 ^bc^	0.9918 ± 0.0002 ^a^
3b	14.85 ± 0.10 ^d^	1.85 ± 0.05 ^ab^	3.71 ± 0.02 ^a^	4.45 ± 0.05 ^c^	0.55 ± 0.01 ^d^	0.85 ± 0.05 ^ab^	0.80 ± 0.05 ^bc^	0.9917 ± 0.0001 ^a^
4b	14.95 ± 0.10 ^de^	1.70 ± 0.05 ^a^	3.71 ± 0.02 ^a^	4.50 ± 0.05 ^c^	0.58 ± 0.01 ^e^	0.80 ± 0.05 ^a^	0.90 ± 0.05 ^cd^	0.9915 ± 0.0001 ^a^
1c	14.30 ± 0.05 ^b^	2.00 ± 0.05 ^bc^	3.71 ± 0.02 ^a^	4.35 ± 0.05 ^bc^	0.52 ± 0.01 ^c^	1.00 ± 0.05 ^b^	0.70 ± 0.05 ^b^	0.9917 ± 0.0001 ^a^
2c	14.40 ± 0.10 ^b^	1.95 ± 0.05 ^b^	3.70 ± 0.03 ^a^	4.40 ± 0.05 ^bc^	0.57 ± 0.01 ^de^	1.00 ± 0.05 ^b^	0.75 ± 0.05 ^bc^	0.9918 ± 0.0002 ^a^
3c	14.35 ± 0.05 ^b^	1.90 ± 0.05 ^b^	3.70 ± 0.03 ^a^	4.35 ± 0.05 ^bc^	0.56 ± 0.01 ^d^	1.00 ± 0.05 ^b^	0.80 ± 0.05 ^bc^	0.9916 ± 0.0001 ^a^
4c	14.85 ± 0.05 ^d^	1.85 ± 0.05 ^ab^	3.69 ± 0.03 ^a^	4.50 ± 0.05 ^c^	0.58 ± 0.01 ^e^	0.85 ± 0.05 ^ab^	0.90 ± 0.05 ^cd^	0.9916 ± 0.0001 ^a^
1d	14.30 ± 0.05 ^b^	2.00 ± 0.05 ^bc^	3.69 ± 0.03 ^a^	4.30 ± 0.05 ^b^	0.50 ± 0.01 ^bc^	1.05 ± 0.05 ^b^	0.80 ± 0.05 ^bc^	0.9915 ± 0.0001 ^a^
2d	14.25 ± 0.05 ^ab^	2.00 ± 0.05 ^bc^	3.70 ± 0.02 ^a^	4.45 ± 0.05 ^c^	0.54 ± 0.01 ^cd^	1.00 ± 0.05 ^b^	0.80 ± 0.05 ^bc^	0.9918 ± 0.0001 ^a^
3d	14.65 ± 0.05 ^c^	1.85 ± 0.05 ^ab^	3.69 ± 0.02 ^a^	4.45 ± 0.05 ^c^	0.55 ± 0.01 ^d^	0.95 ± 0.05 ^b^	0.75 ± 0.05 ^bc^	0.9917 ± 0.0001 ^a^
4d	14.95 ± 0.10 ^de^	1.95 ± 0.05 ^b^	3.70 ± 0.01 ^a^	4.50 ± 0.05 ^c^	0.57 ± 0.01 ^de^	0.80 ± 0.05 ^a^	0.95 ± 0.05 ^cd^	0.9914 ± 0.0002 ^a^
1e	14.20 ± 0.05 ^ab^	1.85 ± 0.05 ^ab^	3.71 ± 0.02 ^a^	4.25 ± 0.05 ^b^	0.51 ± 0.01 ^c^	1.00 ± 0.05 ^bc^	0.80 ± 0.05 ^bc^	0.9918 ± 0.0001 ^a^
2e	14.25 ± 0.05 ^ab^	1.75 ± 0.05 ^ab^	3.68 ± 0.02 ^a^	4.55 ± 0.05 ^cd^	0.57 ± 0.01 ^de^	0.95 ± 0.05 ^b^	0.90 ± 0.05 ^cd^	0.9916 ± 0.0001 ^a^
3e	14.40 ± 0.05 ^b^	1.70 ± 0.05 ^a^	3.71 ± 0.02 ^a^	4.55 ± 0.05 ^cd^	0.55 ± 0.01 ^d^	0.95 ± 0.05 ^b^	0.85 ± 0.05 ^c^	0.9918 ± 0.0001 ^a^
4e	15.05 ± 0.05 ^e^	1.60 ± 0.05 ^a^	3.69 ± 0.02 ^a^	4.65 ± 0.05 ^d^	0.60 ± 0.01 ^e^	0.80 ± 0.05 ^a^	1.00 ± 0.05 ^d^	0.9917 ± 0.0002 ^a^

Significant differences (*p* < 0.05) in the same column have been marked with different superscript letters (a–e). Me20—Merlot vintage 2020 sample before ageing; a—stainless-steel tank; b—Excellence wooden barrel with medium toasting; c—Excellence wooden barrel with medium-plus toasting; d—Excellence wooden barrel with medium-long toasting; e—Premium wooden barrel with medium toasting; 1a–1e—sampling in June 2021; 2a–2e—sampling in September 2021; 3a–3e—sampling in December 2021; 4a–4e—sampling in March 2022.

**Table 3:** Chemical composition of Merlot vintage 2021 and samples obtained during 12-month ageing in different vessels.

Sample	Ethanol (vol.%)	Total Sugar (g/L)	pH	Total Acidity (g/L)	Volatile Acidity (g/L)	Malic Acid (g/L)	Lactic Acid (g/L)	Density (kg/L)
Me21	13.95 ± 0.05 ^ab^	1.95 ± 0.05 ^d^	3.65 ± 0.01 ^a^	4.10 ± 0.05 ^bc^	0.52 ± 0.01 ^b^	1.15 ± 0.05 ^c^	0.50 ± 0.05 ^ab^	0.9916 ± 0.0001 ^a^
1A	13.75 ± 0.05 ^a^	1.90 ± 0.05 ^d^	3.64 ± 0.01 ^a^	4.05 ± 0.05 ^b^	0.51 ± 0.01 ^b^	1.15 ± 0.05 ^c^	0.45 ± 0.05 ^a^	0.9918 ± 0.0002 ^a^
2A	13.75 ± 0.15 ^a^	1.75 ± 0.05 ^bc^	3.64 ± 0.02 ^a^	4.10 ± 0.05 ^bc^	0.53 ± 0.01 ^bc^	0.90 ± 0.05 ^ab^	0.40 ± 0.05 ^a^	0.9917 ± 0.0001 ^a^
3A	13.90 ± 0.10 ^ab^	1.80 ± 0.05 ^cd^	3.65 ± 0.02 ^a^	3.90 ± 0.05 ^a^	0.51 ± 0.01 ^b^	0.90 ± 0.05 ^ab^	0.55 ± 0.05 ^a^	0.9918 ± 0.0001 ^a^
4A	14.00 ± 0.10 ^b^	1.85 ± 0.05 ^cd^	3.68 ± 0.03 ^a^	4.55 ± 0.05 ^ef^	0.54 ± 0.01 ^bc^	0.90 ± 0.05 ^ab^	0.60 ± 0.05 ^b^	0.9918 ± 0.0002 ^a^
1B	13.95 ± 0.05 ^ab^	1.75 ± 0.05 ^bc^	3.63 ± 0.02 ^a^	4.20 ± 0.05 ^c^	0.54 ± 0.01 ^bc^	1.00 ± 0.05 ^b^	0.50 ± 0.05 ^ab^	0.9917 ± 0.0001 ^a^
2B	13.80 ± 0.10 ^ab^	1.70 ± 0.05 ^b^	3.64 ± 0.02 ^a^	4.10 ± 0.05 ^bc^	0.55 ± 0.01 ^c^	0.85 ± 0.05 ^a^	0.40 ± 0.05 ^a^	0.9916 ± 0.0001 ^a^
3B	14.05 ± 0.05 ^bc^	1.75 ± 0.05 ^bc^	3.65 ± 0.02 ^a^	4.60 ± 0.05 ^f^	0.59 ± 0.01 ^d^	0.90 ± 0.05 ^ab^	0.65 ± 0.05 ^b^	0.9916 ± 0.0002 ^a^
4B	14.05 ± 0.05 ^bc^	1.80 ± 0.05 ^c^	3.65 ± 0.02 ^a^	4.45 ± 0.05 ^de^	0.58 ± 0.01 ^cd^	0.85 ± 0.05 ^a^	0.70 ± 0.05 ^c^	0.9918 ± 0.0002 ^a^
1C	14.05 ± 0.05 ^bc^	1.80 ± 0.05 ^c^	3.69 ± 0.03 ^a^	3.90 ± 0.05 ^a^	0.53 ± 0.01 ^bc^	1.00 ± 0.05 ^b^	0.45 ± 0.05 ^a^	0.9915 ± 0.0003 ^a^
2C	14.25 ± 0.05 ^cd^	1.75 ± 0.05 ^bc^	3.68 ± 0.03 ^a^	4.30 ± 0.05 ^cd^	0.58 ± 0.01 ^d^	0.90 ± 0.05 ^ab^	0.60 ± 0.05 ^b^	0.9914 ± 0.0001 ^a^
3C	14.25 ± 0.05 ^cd^	1.65 ± 0.05 ^ab^	3.65 ± 0.02 ^a^	4.10 ± 0.05 ^bc^	0.57 ± 0.01 ^cd^	0.95 ± 0.05 ^ab^	0.55 ± 0.05 ^ab^	0.9917 ± 0.0001 ^a^
4C	14.35 ± 0.05 ^d^	1.65 ± 0.05 ^ab^	3.66 ± 0.02 ^a^	4.40 ± 0.05 ^d^	0.55 ± 0.01 ^c^	0.95 ± 0.05 ^ab^	0.80 ± 0.05 ^c^	0.9916 ± 0.0002 ^a^
1D	14.10 ± 0.10 ^bc^	1.70 ± 0.05 ^b^	3.67 ± 0.02 ^a^	4.25 ± 0.05 ^c^	0.56 ± 0.01 ^cd^	1.15 ± 0.05 ^c^	0.55 ± 0.05 ^ab^	0.9917 ± 0.0001 ^a^
2D	14.00 ± 0.10 ^bc^	1.60 ± 0.05 ^ab^	3.65 ± 0.02 ^a^	4.25 ± 0.05 ^c^	0.51 ± 0.01 ^b^	1.10 ± 0.05 ^bc^	0.45 ± 0.05 ^a^	0.9914 ± 0.0001 ^a^
3D	14.05 ± 0.05 ^bc^	1.60 ± 0.05 ^ab^	3.64 ± 0.02 ^a^	4.15 ± 0.05 ^bc^	0.52 ± 0.01 ^b^	0.85 ± 0.05 ^a^	0.50 ± 0.05 ^ab^	0.9919 ± 0.0005 ^a^
4D	14.15 ± 0.15 ^bc^	1.60 ± 0.05 ^ab^	3.63 ± 0.03 ^a^	4.35 ± 0.05 ^cd^	0.59 ± 0.01 ^d^	0.85 ± 0.05 ^a^	0.65 ± 0.05 ^b^	0.9919 ± 0.0006 ^a^
1E	14.05 ± 0.15 ^bc^	1.65 ± 0.05 ^ab^	3.63 ± 0.03 ^a^	3.90 ± 0.05 ^a^	0.45 ± 0.01 ^a^	1.10 ± 0.05 ^bc^	0.45 ± 0.05 ^a^	0.9919 ± 0.0001 ^a^
2E	14.20 ± 0.20 ^cd^	1.55 ± 0.05 ^a^	3.66 ± 0.03 ^a^	4.40 ± 0.05 ^d^	0.54 ± 0.01 ^bc^	0.95 ± 0.05 ^ab^	0.55 ± 0.05 ^ab^	0.9915 ± 0.0001 ^a^
3E	14.20 ± 0.10 ^cd^	1.50 ± 0.05 ^a^	3.64 ± 0.01 ^a^	4.05 ± 0.05 ^b^	0.54 ± 0.01 ^bc^	0.85 ± 0.05 ^a^	0.55 ± 0.05 ^ab^	0.9916 ± 0.0001 ^a^
4E	14.50 ± 0.20 ^d^	1.50 ± 0.05 ^a^	3.67 ± 0.02 ^a^	4.50 ± 0.05 ^d^	0.57 ± 0.01 ^cd^	0.80 ± 0.05 ^a^	0.95 ± 0.05 ^d^	0.9916 ± 0.0001 ^a^

Significant differences (*p* < 0.05) in the same column have been marked with different superscript letters (a–f). Me21—Merlot vintage 2021 sample before ageing; A—stainless-steel tank; B—Excellence wooden barrel with medium toasting; C—Excellence wooden barrel with medium-plus toasting; D—Excellence wooden barrel with medium-long toasting; E—Premium wooden barrel with medium toasting; 1A–1E—sampling in August 2022; 2A–2E—sampling in November 2022; 3A–3E—sampling in February 2023; 4A–4E—sampling in May 2023.

The parameters include ethanol and total sugar content, pH, total and volatile acidity, concentrations of malic and lactic acids, and density. The chemical profiles of both vintages were largely similar, with only minor variations. The 2020 vintage (Me20) showed slightly higher ethanol and sugar contents (14.20 vol.% and 2.10 g/L, respectively) compared to the 2021 vintage Merlot (Me21), which had 13.95 vol.% ethanol and 1.95 g/L sugar. During the 12-month ageing period, ethanol content increased marginally across all storage vessels for both vintages compared to the initial measurements. Conversely, total sugar content showed a slight reduction in all samples over the same period. The lowest sugar concentration was recorded in PMT samples, with 1.60 g/L for Me20 and 1.50 g/L for Me21.

Density remained consistent during ageing across all vessels, with an average value of 0.9916 kg/L for both vintages. The same was observed for pH; no significant change between samples obtained after ageing, but slightly higher pH, was measured in samples from vintage 2020 (average was 3.71) than in the ones from 2021 (average was 3.65). Total acidity showed a slight increase after 12 months of ageing in all vessels for both vintages, rising from the initial values of 4.05 g/L in Me20 and 4.10 g/L in Me21. The highest total acidity after 12 months was recorded in PMT for the 2020 vintage (4.65 g/L) and in EMLT for the 2021 vintage (5.35 g/L). Volatile acidity also exhibited a gradual increase during ageing across all samples, relative to initial values. For malic and lactic acids, a slight reduction in malic acid and a corresponding increase in lactic acid concentrations were noted during the 12-month ageing period across all vessels. The lowest malic acid concentrations were observed at the end of ageing in both vintages, with no significant differences between vessels. In contrast, differences in lactic acid concentrations were minimal, except for PMT samples, where the highest concentrations were recorded (1.00 g/L for Me20 and 0.95 g/L for Me21).

### 3.2. Phenolic Profile and Antioxidant Activity

Polyphenol content (TPC), flavonoid content (TFC), anthocyanins (MAC), tannins (CTC and HTC), polymeric colour (PC), and antioxidant activity (DPPH, ABTS, FRAP, and CUPRAC assays) were measured spectrophotometrically. The results are shown in Table 4 and Table 5. 

**Table 4:** Total polyphenols, flavonoids, monomeric anthocyanins, condensed and hydrolysable tannin content, polymeric colour percentage, and antioxidant activity (DPPH, ABTS, FRAP, and CUPRAC method) in 2020 vintage Merlot and samples obtained after 12 months of ageing in different vessels.

Sample	TPC (mg/L)	TFC (mg/L)	MAC (mg/L)	PC (%)	CTC (mg/L)	HTC (mg/L)	DPPH (μmol/100 mL)	ABTS (μmol/100 mL)	FRAP (μmol/100 mL)	CUPRAC (μmol/100 mL)
Me20	2.23 ± 0.02 ^a^	1.00 ± 0.02 ^b^	79.36 ± 0.48 ^i^	61.04 ± 0.76 ^a^	495.28 ± 1.84 ^j^	-	3.95 ± 0.16 ^a^	17.42 ± 0.41 ^a^	18.14 ± 0.33 ^f^	50.49 ± 0.16 ^b^
1a	2.35 ± 0.03 ^bc^	1.05 ± 0.02 ^cd^	73.42 ± 1.05 ^h^	63.46 ± 0.26 ^b^	452.29 ± 2.98 ^i^	-	4.45 ± 0.18 ^b^	17.25 ± 0.19 ^a^	23.42 ± 0.26 ^h^	82.01 ± 1.55 ^e^
2a	2.25 ± 0.03 ^ab^	1.06 ± 0.02 ^cd^	72.29 ± 0.04 ^h^	63.33 ± 1.00 ^b^	458.31 ± 4.73 ^i^	-	4.38 ± 0.16 ^b^	17.23 ± 0.25 ^a^	22.16 ± 0.50 ^h^	78.16 ± 1.55 ^de^
3a	2.32 ± 0.02 ^b^	1.08 ± 0.01 ^d^	71.36 ± 0.15 ^h^	64.77 ± 0.54 ^b^	411.21 ± 3.68 ^f^	-	4.36 ± 0.04 ^b^	16.09 ± 0.10 ^a^	23.02 ± 0.46 ^h^	81.30 ± 0.18 ^e^
4a	2.25 ± 0.02 ^a^	1.02 ± 0.02 ^bc^	66.73 ± 0.28 ^f^	66.72 ± 0.44 ^c^	414.12 ± 4.86 ^f^	-	4.54 ± 0.13 ^b^	17.87 ± 0.06 ^a^	22.35 ± 0.45 ^h^	75.83 ± 1.12 ^d^
1b	2.48 ± 0.01 ^d^	0.95 ± 0.03 ^ab^	61.92 ± 0.51 ^c^	84.58 ± 0.02 ^k^	455.51 ± 2.72 ^i^	71.64 ± 2.62 ^a^	4.27 ± 0.25 ^b^	18.07 ± 0.28 ^ab^	18.29 ± 0.38 ^f^	114.09 ± 0.29 ^g^
2b	2.39 ± 0.03 ^c^	0.95 ± 0.04 ^ab^	61.23 ± 0.28 ^c^	83.64 ± 0.46 ^j^	396.33 ± 1.91 ^e^	70.62 ± 1.36 ^a^	4.04 ± 0.20 ^ab^	20.35 ± 0.32 ^d^	19.49 ± 0.67 ^g^	88.26 ± 0.96 ^ef^
3b	2.38 ± 0.02 ^c^	0.90 ± 0.02 ^a^	61.60 ± 0.73 ^c^	83.96 ± 0.31 ^j^	388.76 ± 2.73 ^d^	74.01 ± 1.54 ^a^	3.80 ± 0.06 ^a^	20.54 ± 0.63 ^de^	21.22 ± 0.76 ^f^	95.80 ± 0.01 ^f^
4b	2.43 ± 0.01 ^c^	1.02 ± 0.02 ^bc^	59.76 ± 0.36 ^b^	85.64 ± 0.01 ^l^	356.13 ± 2.25 ^c^	81.16 ± 2.88 ^b^	4.57 ± 0.25 ^b^	18.69 ± 0.55 ^bc^	22.29 ± 0.26 ^f^	95.40 ± 1.00 ^f^
1c	2.48 ± 0.01 ^d^	1.15 ± 0.02 ^ef^	65.07 ± 0.19 ^e^	81.82 ± 0.55 ^i^	425.62 ± 1.32 ^g^	80.91 ± 3.58 ^b^	7.26 ± 0.33 ^e^	21.77 ± 0.55 ^e^	16.65 ± 0.38 ^e^	77.81 ± 0.84 ^de^
2c	2.47 ± 0.01 ^d^	1.17 ± 0.02 ^f^	61.18 ± 0.49 ^c^	84.61 ± 0.32 ^j^	419.69 ± 4.07 ^fg^	92.72 ± 5.31 ^de^	6.96 ± 0.21 ^e^	23.57 ± 0.09 ^g^	14.52 ± 0.06 ^d^	69.04 ± 0.93 ^d^
3c	2.42 ± 0.02 ^c^	1.15 ± 0.01 ^ef^	59.07 ± 0.42 ^b^	83.89 ± 0.13 ^j^	359.41 ± 3.91 ^c^	98.72 ± 3.14 ^e^	7.03 ± 0.11 ^e^	22.45 ± 0.09 ^f^	16.00 ± 0.62 ^e^	74.64 ± 0.44 ^d^
4c	2.46 ± 0.01 ^d^	1.14 ± 0.01 ^e^	56.33 ± 0.81 ^a^	83.67 ± 0.95 ^j^	327.03 ± 3.56 ^a^	97.20 ± 1.98 ^e^	6.48 ± 0.18 ^d^	22.29 ± 0.09 ^ef^	13.27 ± 0.52 ^c^	57.59 ± 0.69 ^c^
1d	2.46 ± 0.03 ^d^	1.05 ± 0.02 ^c^	63.65 ± 0.63 ^d^	77.46 ± 0.12 ^h^	438.62 ± 8.10 ^h^	87.94 ± 1.96 ^cd^	5.73 ± 0.25 ^c^	19.93 ± 0.09 ^c^	14.23 ± 0.10 ^d^	84.99 ± 0.60 ^e^
2d	2.53 ± 0.02 ^f^	0.99 ± 0.03 ^b^	67.86 ± 0.85 ^fg^	75.96 ± 0.33 ^g^	386.72 ± 9.02 ^d^	83.61 ± 2.95 ^bc^	5.85 ± 0.43 ^c^	19.21 ± 0.19 ^c^	12.96 ± 0.32 ^b^	80.32 ± 1.21 ^e^
3d	2.48 ± 0.02 ^d^	1.11 ± 0.02 ^de^	62.75 ± 0.92 ^cd^	77.46 ± 0.01 ^h^	348.05 ± 6.21 ^bc^	90.93 ± 2.72 ^d^	5.97 ± 0.17 ^c^	20.15 ± 0.09 ^d^	13.68 ± 0.13 ^c^	71.83 ± 0.90 ^d^
4d	2.42 ± 0.01 ^c^	1.02 ± 0.03 ^bc^	56.90 ± 0.57 ^a^	83.12 ± 0.55 ^j^	339.27 ± 4.06 ^b^	91.13 ± 2.16 ^d^	6.29 ± 0.12 ^d^	20.57 ± 0.09 ^d^	10.37 ± 0.17 ^a^	59.02 ± 0.55 ^c^
1e	2.31 ± 0.02 ^b^	1.07 ± 0.02 ^cd^	70.36 ± 0.50 ^h^	70.59 ± 0.23 ^d^	432.85 ± 2.15 ^h^	76.01 ± 2.66 ^ab^	6.07 ± 0.28 ^cd^	17.56 ± 0.09 ^a^	12.05 ± 0.49 ^b^	46.66 ± 0.85 ^a^
2e	2.30 ± 0.02 ^b^	1.09 ± 0.02 ^d^	71.10 ± 0.60 ^h^	70.96 ± 0.24 ^d^	419.60 ± 2.43 ^fg^	90.74 ± 2.25 ^d^	5.86 ± 0.28 ^c^	18.37 ± 0.09 ^b^	13.25 ± 0.37 ^bc^	50.90 ± 0.57 ^b^
3e	2.29 ± 0.01 ^b^	1.06 ± 0.01 ^cd^	68.33 ± 0.13 ^g^	72.07 ± 0.08 ^e^	398.81 ± 0.90 ^e^	97.12 ± 3.51 ^e^	5.73 ± 0.26 ^c^	18.50 ± 0.09 ^b^	13.54 ± 0.05 ^c^	51.06 ± 0.35 ^b^
4e	2.39 ± 0.03 ^c^	1.07 ± 0.00 ^cd^	64.61 ± 0.20 ^de^	74.21 ± 0.35 ^f^	380.08 ± 3.72 ^d^	109.03 ± 4.58 ^f^	5.65 ± 0.11 ^c^	19.81 ± 0.09 ^c^	12.48 ± 0.32 ^b^	47.19 ± 0.96 ^a^

Significant differences (*p* < 0.05) in the same column have been marked with different superscript letters (a–l). Me20—Merlot vintage 2020 sample before ageing; a—stainless-steel tank; b—Excellence wooden barrel with medium toasting; c—Excellence wooden barrel with medium-plus toasting; d—Excellence wooden barrel with medium-long toasting; e—Premium wooden barrel with medium toasting; 1a–1e—sampling in June 2021; 2a–2e—sampling in September 2021; 3a–3e—sampling in December 2021; 4a–4e—sampling in March 2022.

**Table 5:** Total polyphenols, flavonoids, monomeric anthocyanins, condensed and hydrolysable tannin content, polymeric colour percentage, and antioxidant activity (DPPH, ABTS, FRAP, and CUPRAC method) in 2021 vintage Merlot and samples obtained after 12 months of ageing in different vessels.

Sample	TPC (mg/L)	TFC (mg/L)	MAC (mg/L)	PC (%)	CTC (mg/L)	HTC (mg/L)	DPPH (μmol/100 mL)	ABTS (μmol/100 mL)	FRAP (μmol/100 mL)	CUPRAC (μmol/100 mL)
Me21	2.68 ± 0.01 ^b^	1.39 ± 0.01 ^a^	112.87 ± 0.46 ^m^	53.77 ± 0.20 ^a^	735.54 ± 4.58 ^i^	-	7.86 ± 0.29 ^a^	21.00 ± 0.42 ^a^	9.46 ± 0.33 ^a^	125.83 ± 0.60 ^e^
1A	2.74 ± 0.01 ^c^	1.37 ± 0.01 ^a^	88.63 ± 0.82 ^e^	57.45 ± 0.88 ^d^	722.94 ± 4.31 ^h^	-	10.82 ± 0.31 ^c^	22.87 ± 0.25 ^c^	9.93 ± 0.01 ^a^	177.90 ± 0.15 ^j^
2A	2.69 ± 0.02 ^b^	1.44 ± 0.01 ^b^	91.81 ± 0.67 ^g^	54.19 ± 0.60 ^ab^	692.40 ± 4.33 ^de^	-	10.26 ± 0.29 ^c^	21.66 ± 0.64 ^ab^	17.56 ± 0.26 ^c^	135.65 ± 0.44 ^f^
3A	2.81 ± 0.02 ^cd^	1.44 ± 0.01 ^b^	91.65 ± 0.36 ^g^	55.61 ± 0.81 ^b^	695.96 ± 2.00 ^e^	-	12.50 ± 0.21 ^e^	22.16 ± 0.16 ^b^	17.05 ± 0.30 ^c^	103.63 ± 0.56 ^c^
4A	2.81 ± 0.02 ^cd^	1.44 ± 0.01 ^b^	89.50 ± 0.39 ^e^	57.43 ± 0.84 ^d^	691.86 ± 3.77 ^de^	-	12.83 ± 0.24 ^e^	22.48 ± 0.18 ^bc^	20.28 ± 0.37 ^ef^	103.95 ± 0.58 ^c^
1B	2.85 ± 0.02 ^de^	1.54 ± 0.01 ^d^	86.54 ± 1.06 ^d^	58.50 ± 0.14 ^e^	723.36 ± 1.02 ^h^	147.52 ± 6.48 ^a^	9.53 ± 0.34 ^b^	23.84 ± 0.28 ^d^	9.07 ± 0.13 ^a^	196.83 ± 0.40 ^l^
2B	2.90 ± 0.01 ^e^	1.58 ± 0.01 ^e^	90.96 ± 0.53 ^f^	56.01 ± 0.78 ^bc^	697.71 ± 2.58 ^e^	140.78 ± 3.60 ^a^	10.46 ± 0.21 ^c^	29.07 ± 0.13 ^h^	16.08 ± 0.13 ^b^	175.71 ± 0.45 ^j^
3B	2.84 ± 0.02 ^de^	1.47 ± 0.01 ^bc^	83.64 ± 0.94 ^bc^	56.67 ± 0.11 ^c^	685.25 ± 3.96 ^d^	210.52 ± 3.03 ^de^	10.78 ± 0.39 ^cd^	24.35 ± 0.30 ^d^	16.94 ± 0.43 ^bc^	89.78 ± 0.18 ^a^
4B	2.84 ± 0.02 ^de^	1.47 ± 0.01 ^bc^	81.48 ± 0.10 ^a^	61.20 ± 0.26 ^g^	681.78 ± 3.60 ^cd^	217.65 ± 5.85 ^e^	11.60 ± 0.41 ^d^	25.17 ± 0.33 ^e^	20.18 ± 0.65 ^ef^	90.60 ± 0.20 ^a^
1C	2.79 ± 0.03 ^cd^	1.46 ± 0.01 ^bc^	102.34 ± 0.40 ^j^	59.11 ± 0.83 ^ef^	714.07 ± 3.03 ^g^	204.88 ± 2.58 ^d^	15.51 ± 0.17 ^g^	30.70 ± 0.15 ^i^	19.55 ± 0.38 ^e^	161.78 ± 0.18 ^i^
2C	2.88 ± 0.02 ^e^	1.59 ± 0.01 ^e^	94.66 ± 0.89 ^i^	60.20 ± 0.60 ^f^	695.60 ± 1.27 ^e^	218.87 ± 1.36 ^e^	14.08 ± 0.06 ^f^	33.49 ± 0.05 ^j^	20.01 ± 0.13 ^e^	145.19 ± 0.97 ^g^
3C	2.81 ± 0.02 ^cd^	1.49 ± 0.01 ^c^	87.68 ± 1.77 ^de^	60.86 ± 0.42 ^fg^	682.99 ± 1.37 ^d^	217.23 ± 2.31 ^e^	12.14 ± 0.50 ^de^	27.98 ± 0.45 ^fg^	15.93 ± 0.12 ^b^	108.25 ± 0.45 ^d^
4C	2.81 ± 0.02 ^cd^	1.49 ± 0.01 ^c^	85.52 ± 0.19 ^d^	63.69 ± 0.45 ^h^	657.15 ± 2.57 ^a^	259.71 ± 8.69 ^g^	12.46 ± 0.52 ^de^	28.31 ± 0.47 ^g^	19.16 ± 0.41 ^de^	108.57 ± 0.48 ^d^
1D	2.80 ± 0.02 ^d^	1.52 ± 0.01 ^cd^	105.81 ± 1.31 ^k^	55.52 ± 0.73 ^b^	691.72 ± 4.41 ^de^	192.01 ± 4.37 ^c^	14.53 ± 0.32 ^fg^	29.12 ± 0.03 ^h^	17.28 ± 0.15 ^c^	191.35 ± 0.41 ^k^
2D	2.77 ± 0.01 ^c^	1.56 ± 0.02 ^de^	96.24 ± 0.49 ^i^	59.80 ± 0.53 ^f^	681.44 ± 5.73 ^cd^	197.94 ± 5.35 ^cd^	14.11 ± 0.16 ^f^	27.36 ± 0.16 ^f^	16.88 ± 0.40 ^bc^	144.71 ± 0.28 ^g^
3D	2.82 ± 0.02 ^d^	1.40 ± 0.02 ^ab^	84.23 ± 0.71 ^c^	60.12 ± 0.53 ^f^	666.55 ± 3.26 ^b^	194.25 ± 5.80 ^c^	12.07 ± 0.26 ^de^	21.97 ± 0.12 ^b^	16.56 ± 0.10 ^b^	98.94 ± 0.56 ^b^
4D	2.82 ± 0.02 ^d^	1.41 ± 0.02 ^ab^	82.08 ± 0.08 ^b^	61.44 ± 0.55 ^g^	666.64 ± 2.52 ^b^	209.29 ± 4.54 ^de^	12.39 ± 0.29 ^de^	22.30 ± 0.15 ^b^	19.80 ± 0.28 ^e^	99.27 ± 0.58 ^b^
1E	2.56 ± 0.02 ^a^	1.45 ± 0.03 ^bc^	104.88 ± 0.87 ^k^	55.16 ± 0.50 ^b^	702.77 ± 2.46 ^f^	183.72 ± 2.28 ^b^	12.20 ± 0.21 ^de^	29.45 ± 0.59 ^h^	17.58 ± 0.16 ^c^	150.29 ± 0.23 ^h^
2E	2.80 ± 0.02 ^d^	1.46 ± 0.01 ^bc^	109.95 ± 0.23 ^l^	55.92 ± 0.61 ^bc^	705.62 ± 3.62 ^f^	202.94 ± 1.73 ^d^	15.23 ± 0.48 ^f^	28.37 ± 0.34 ^g^	18.46 ± 0.37 ^d^	144.67 ± 0.45 ^g^
3E	2.78 ± 0.01 ^c^	1.47 ± 0.01 ^bc^	93.03 ± 0.41 ^h^	57.68 ± 0.18 ^d^	674.72 ± 3.70 ^c^	202.71 ± 5.44 ^d^	11.63 ± 0.24 ^d^	25.66 ± 0.63 ^e^	18.13 ± 0.48 ^cd^	125.96 ± 0.92 ^e^
4E	2.78 ± 0.01 ^c^	1.47 ± 0.01 ^bc^	90.88 ± 0.04 ^f^	60.00 ± 0.21 ^f^	662.06 ± 5.41 ^ab^	222.84 ± 1.18 ^f^	13.95 ± 0.26 ^f^	27.98 ± 0.66 ^fg^	21.37 ± 0.66 ^f^	128.28 ± 0.94 ^e^

Significant differences (*p* < 0.05) in the same column have been marked with different superscript letters (a–m). Me21—Merlot vintage 2021 sample before ageing; A—stainless-steel tank; B—Excellence wooden barrel with medium toasting; C—Excellence wooden barrel with medium-plus toasting; D—Excellence wooden barrel with medium-long toasting; E—Premium wooden barrel with medium toasting; 1A–1E—sampling in August 2022; 2A–2E—sampling in November 2022; 3A–3E—sampling in February 2023; 4A–4E—sampling in May 2023.

The 2021 Merlot vintage and its aged samples exhibited slightly higher TPC, TFC, MAC, CTC, HTC, and antioxidant activity compared to the 2020 vintage. This was consistent across all vessels and antioxidant assays (DPPH, ABTS, and CUPRAC).

The initial Me20 contained 2.23 g/L of TPC and 1.00 g/L of TFC, while Me21 had 2.68 g/L and 1.39 g/L, respectively. After 12 months of ageing, the highest TPC and TFC in Me20 were measured in the EMT+ barrel (2.46 and 1.14 g/L, respectively). In contrast, no significant difference was found between the initial TPC value in Me20 and that measured after 12 months of ageing in SST (2.25 g/L). For Me21, the EMT+, EMT, and PMT barrels were the most favourable for TFC, with concentrations reaching 1.47–1.49 g/L after 12 months. The average final TPC for Me21 across all vessels was the highest (2.82 g/L), except for PMT, which had the lowest TPC after ageing (2.78 g/L).

The highest concentrations of total monomeric anthocyanins (MAC) were found in the initial wines, with Me20 at 79.36 mg/L and Me21 at 112.87 mg/L. After 12 months of ageing, a decrease in MAC content was observed in all vessels compared to the initial values. The lowest MAC in the 2020 vintage was found in the EMT+ and EMLT barrels (approximately 56 mg/L in both). In the 2021 vintage, the lowest MAC after ageing was observed in the EMT barrel (81.48 mg/L). Polymeric colour (PC) increased during the ageing process, with the lowest PC values measured in the initial wines: 61.04% for Me20 and 53.77% for Me21. After 12 months of ageing, the highest PC for Me20 was observed in the EMT barrel (85.64%), while the highest PC for Me21 was found in the EMT+ barrel (63.69%).

The concentrations of condensed tannins (proanthocyanidins) in the initial wines were 495.28 mg/L (2020 vintage) and 735.54 mg/L (2021 vintage). During 12 months of ageing, condensed tannins decreased in almost all vessels for both vintages. The longer the storage, the lower the content of condensed tannins. However, slight differences could be observed between different vessels. After 12 months of ageing, the retention of condensed tannins was the highest in both 2020 and 2021 vintages of Merlot wine that had aged in SST (83.6 and 94.1%, respectively). On the other hand, the decrease in CTC was more pronounced in wooden barrels, with their highest reduction of 33.9% in 2020 Merlot from the EMT+ barrel and of 9.9% in 2021 Merlot from the PMT barrel, compared to the corresponding initial wine.

In the same table, hydrolysable tannin content was presented. It could be noted that hydrolysable tannins were not detected in the initial wines or the samples obtained from SST. In the Merlot wines obtained from wooden barrels with different toasting methods, different concentrations of hydrolysable tannins were measured. First, the 2021 Merlot from wooden barrels contained higher concentrations than the 2020 Merlot from the same barrels with the same ageing time. However, in all barrels and both wine vintages, an increasing trend of hydrolysable tannin content during 12 months of ageing could be observed. The lowest concentrations were measured in the EMT barrel during the first 3 and 6 months of ageing (an average of 71.13 mg/L in 2020 Merlot and 144.15 mg/L in 2021 Merlot). After 12 months of ageing, the highest concentrations of hydrolysable tannins were measured in PMT barrel for 2020 Merlot (109.03 mg/L) and in EMT+ and PMT barrel for 2021 Merlot (259.71 and 222.84 mg/L, respectively).

The antioxidant activity determined by DPPH and ABTS assays among Me20 samples followed a similar trend as the TPC and TFC in those samples: after 12 months of ageing, the antioxidant increased, with the highest value measured in the EMT+ barrel (6.48 and 22.29 μmol/100 mL, respectively). In the same barrel, after 12 months of ageing, the highest antioxidant activity determined by ABTS among Me21 samples was measured, 28.31 μmol/100 mL. In other vessels, it was also higher than the initial ABTS antioxidant activity (21.00 μmol/100 mL) after 12 months of ageing. The DPPH assay also showed an increase in antioxidant activity during the 12-month ageing of 2021 Merlot, with the highest value measured in the PMT barrel (13.95 μmol/100 mL). The PMT barrel also resulted in the highest antioxidant activity determined by FRAP and CUPRAC (21.37 and 128.28 μmol/100 mL, respectively) after 12 months of the ageing of the 2021 Merlot. On the other hand, 12 months of the ageing of the 2020 Merlot in the same barrel resulted in the lowest CUPRAC antioxidant activity (47.19 μmol/100 mL). In the rest of the vessels, after the full ageing period, CUPRAC activity in Me20 wine was higher than the initial one (50.49 μmol/100 mL). The lowest FRAP antioxidant activity of Me20 wine was measured in EMLT (10.37 μmol/100 mL). However, the FRAP activity increased during the 12-month ageing of Me20 in SST and EMT, compared to the initial one (18.14 μmol/100 mL).

To gain a deeper understanding of the phenolic profile of the two Merlot vintages and the samples aged in different vessels, the main individual phenolic compounds in the Merlot wines were determined. These included two flavonols (quercetin and hyperoside), gallic acid as a representative of hydroxybenzoic acids, four hydroxycinnamic acids (p-coumaric, caftaric, coutaric, and caffeic acid), two flavan-3-ols ((+)-catechin and (−)-epicatechin), and two anthocyanins (malvidin-3-glucoside and delphinidin-3-glucoside). The results are provided in Table 6 and Table 7.

**Table 6:** Individual phenolic compounds (mg/L) in 2020 vintage Merlot and samples obtained after 12 months of ageing in different vessels.

Sample	Quercetin	Hyperoside	Gallic Acid	p-Coumaric Acid	Caftaric Acid	Coutaric Acid	Caffeic Acid	(+)-Catechin	(−)-Epicatechin	Malvidin-3- Glucoside	Delphinidin-3-Glucoside
Me20	10.60 ± 0.03 ^i^	10.22 ± 0.13 ^g^	28.33 ± 0.95 ^i^	6.44 ± 0.22 ^a^	46.68 ± 0.82 ^j^	15.34 ± 0.47 ^g^	8.09 ± 0.21 ^c^	58.36 ± 0.02 ^j^	19.34 ± 0.20 ^e^	10.51 ± 0.08 ^g^	2.68 ± 0.04 ^h^
1a	12.58 ± 0.14 ^k^	10.53 ± 0.08 ^h^	22.32 ± 0.02 ^e^	6.88 ± 0.02 ^a^	40.88 ± 0.01 ^i^	12.61 ± 0.01 ^f^	4.23 ± 0.09 ^a^	58.46 ± 0.33 ^j^	17.47 ± 0.12 ^d^	7.83 ± 0.05 ^f^	2.30 ± 0.05 ^g^
2a	12.30 ± 0.05 ^k^	10.80 ± 0.01 ^i^	22.37 ± 0.07 ^e^	7.22 ± 0.02 ^b^	36.34 ± 0.11 ^h^	11.86 ± 0.04 ^e^	6.57 ± 0.01 ^b^	56.84 ± 0.11 ^i^	16.97 ± 0.33 ^d^	7.74 ± 0.02 ^f^	2.17 ± 0.01 ^f^
3a	11.93 ± 0.12 ^j^	10.60 ± 0.09 ^h^	18.24 ± 0.28 ^b^	6.67 ± 0.10 ^a^	39.31 ± 0.70 ^i^	12.42 ± 0.18 ^f^	4.25 ± 0.13 ^a^	55.78 ± 0.23 ^h^	19.01 ± 0.23 ^e^	7.00 ± 0.04 ^e^	1.93 ± 0.01 ^e^
4a	11.78 ± 0.03 ^j^	10.64 ± 0.06 ^h^	25.39 ± 0.01 ^h^	7.75 ± 0.01 ^c^	29.09 ± 0.02 ^f^	23.56 ± 0.02 ^h^	10.57 ± 0.01 ^d^	53.25 ± 0.13 ^f^	15.22 ± 0.03 ^c^	6.96 ± 0.05 ^e^	2.00 ± 0.05 ^e^
1b	9.33 ± 0.00 ^h^	9.59 ± 0.02 ^e^	17.62 ± 0.04 ^a^	9.29 ± 0.02 ^e^	31.53 ± 0.66 ^g^	25.55 ± 0.53 ^i^	19.76 ± 0.04 ^e^	54.59 ± 0.10 ^g^	17.50 ± 0.12 ^d^	6.47 ± 0.10 ^d^	2.03 ± 0.05 ^e^
2b	7.84 ± 0.02 ^a^	9.27 ± 0.04 ^c^	19.71 ± 0.20 ^c^	9.86 ± 0.04 ^f^	15.82 ± 0.05 ^ab^	6.08 ± 0.02 ^a^	24.36 ± 0.05 ^i^	50.63 ± 0.01 ^d^	14.84 ± 0.02 ^b^	5.57 ± 0.03 ^c^	1.57 ± 0.01 ^b^
3b	7.82 ± 0.03 ^a^	9.33 ± 0.03 ^c^	23.06 ± 0.10 ^f^	9.40 ± 0.03 ^e^	18.58 ± 0.35 ^c^	7.81 ± 0.01 ^b^	23.07 ± 0.08 ^fg^	49.99 ± 0.27 ^cd^	13.96 ± 0.08 ^ab^	5.18 ± 0.05 ^b^	1.57 ± 0.07 ^b^
4b	7.82 ± 0.03 ^a^	9.22 ± 0.07 ^bc^	21.36 ± 0.08 ^d^	9.16 ± 0.15 ^de^	14.81 ± 0.24 ^a^	5.97 ± 0.05 ^a^	23.54 ± 0.05 ^h^	47.55 ± 0.51 ^b^	13.21 ± 0.22 ^a^	4.89 ± 0.02 ^ab^	1.56 ± 0.06 ^b^
1c	10.10 ± 0.02 ^i^	9.73 ± 0.01 ^f^	21.61 ± 0.04 ^d^	10.00 ± 0.02 ^g^	18.42 ± 0.03 ^c^	7.44 ± 0.72 ^b^	23.53 ± 0.05 ^h^	52.75 ± 0.17 ^f^	15.82 ± 0.15 ^c^	6.28 ± 0.05 ^d^	1.72 ± 0.03 ^c^
2c	8.67 ± 0.02 ^f^	9.77 ± 0.01 ^f^	20.94 ± 0.25 ^d^	10.00 ± 0.04 ^g^	18.31 ± 0.04 ^c^	7.87 ± 0.03 ^b^	24.20 ± 0.08 ^i^	51.45 ± 0.07 ^e^	14.68 ± 0.55 ^bc^	5.75 ± 0.01 ^c^	1.62 ± 0.02 ^b^
3c	8.14 ± 0.06 ^c^	9.44 ± 0.01 ^d^	23.13 ± 0.01 ^f^	9.34 ± 0.01 ^e^	18.23 ± 0.01 ^c^	7.82 ± 0.05 ^b^	22.78 ± 0.02 ^g^	49.15 ± 0.01 ^c^	14.01 ± 0.55 ^ab^	5.13 ± 0.01 ^b^	1.54 ± 0.02 ^b^
4c	8.88 ± 0.05 ^g^	9.44 ± 0.01 ^d^	21.49 ± 0.01 ^d^	9.02 ± 0.12 ^d^	16.40 ± 0.06 ^b^	7.24 ± 0.03 ^b^	22.82 ± 0.07 ^g^	46.25 ± 0.25 ^a^	12.77 ± 0.77 ^a^	4.57 ± 0.03 ^a^	1.49 ± 0.01 ^a^
1d	9.04 ± 0.11 ^h^	9.27 ± 0.04 ^c^	18.73 ± 0.28 ^b^	9.26 ± 0.14 ^de^	24.35 ± 0.40 ^e^	9.14 ± 0.13 ^d^	19.78 ± 0.32 ^e^	53.09 ± 0.57 ^f^	15.69 ± 0.15 ^c^	6.30 ± 0.03 ^d^	1.75 ± 0.05 ^c^
2d	8.36 ± 0.04 ^d^	9.45 ± 0.01 ^d^	24.58 ± 0.06 ^g^	8.93 ± 0.04 ^d^	23.09 ± 0.46 ^e^	8.98 ± 0.04 ^d^	20.41 ± 0.07 ^e^	51.36 ± 0.10 ^e^	14.57 ± 0.02 ^b^	6.18 ± 0.17 ^d^	2.12 ± 0.01 ^f^
3d	7.93 ± 0.02 ^b^	9.32 ± 0.02 ^c^	23.53 ± 0.09 ^f^	9.30 ± 0.03 ^e^	18.50 ± 0.05 ^c^	7.47 ± 0.59 ^b^	22.30 ± 0.07 ^g^	50.72 ± 0.04 ^d^	14.24 ± 0.06 ^b^	5.63 ± 0.02 ^c^	1.64 ± 0.03 ^b^
4d	7.99 ± 0.04 ^b^	9.32 ± 0.01 ^c^	21.11 ± 0.09 ^d^	9.15 ± 0.04 ^d^	15.22 ± 0.24 ^a^	5.91 ± 0.17 ^a^	23.20 ± 0.10 ^h^	48.45 ± 0.13 ^b^	13.28 ± 0.25 ^a^	5.19 ± 0.16 ^b^	1.55 ± 0.01 ^ab^
1e	8.64 ± 0.03 ^f^	9.10 ± 0.06 ^a^	21.02 ± 0.15 ^d^	10.11 ± 0.07 ^g^	16.08 ± 0.11 ^b^	6.40 ± 0.04 ^a^	26.00 ± 0.66 ^j^	50.87 ± 0.39 ^de^	14.45 ± 0.05 ^b^	6.29 ± 0.01 ^d^	1.82 ± 0.04 ^d^
2e	8.52 ± 0.01 ^e^	9.49 ± 0.01 ^d^	19.40 ± 0.05 ^c^	9.05 ± 0.10 ^d^	23.00 ± 0.05 ^e^	8.77 ± 0.05 ^cd^	20.39 ± 0.04 ^e^	50.44 ± 0.26 ^d^	14.39 ± 0.54 ^b^	5.97 ± 0.22 ^cd^	1.71 ± 0.04 ^c^
3e	8.67 ± 0.06 ^f^	9.61 ± 0.04 ^e^	21.84 ± 0.22 ^de^	9.27 ± 0.06 ^d^	21.51 ± 0.17 ^d^	8.26 ± 0.02 ^c^	21.59 ± 0.17 ^f^	49.80 ± 0.11 ^c^	13.32 ± 0.23 ^a^	5.62 ± 0.17 ^c^	1.57 ± 0.03 ^b^
4e	8.59 ± 0.07 ^e^	9.37 ± 0.03 ^c^	17.79 ± 0.29 ^a^	8.98 ± 0.14 ^d^	20.17 ± 0.36 ^d^	7.90 ± 0.13 ^b^	21.30 ± 0.36 ^f^	48.13 ± 0.39 ^b^	12.89 ± 0.47 ^a^	4.98 ± 0.16 ^ab^	1.47 ± 0.03 ^a^

Significant differences (*p* < 0.05) in the same column have been marked with different superscript letters (a–k). Me20—Merlot vintage 2020 sample before ageing; a—stainless-steel tank; b—Excellence wooden barrel with medium toasting; c—Excellence wooden barrel with medium-plus toasting; d—Excellence wooden barrel with medium-long toasting; e—Premium wooden barrel with medium toasting; 1a–1e—sampling in June 2021; 2a–2e—sampling in September 2021; 3a–3e—sampling in December 2021; 4a–4e—sampling in March 2022.

**Table 7:** Individual phenolic compounds (mg/L) in 2021 vintage Merlot and samples obtained after 12 months of ageing in different vessels.

Sample	Quercetin	Hyperoside	Gallic Acid	p-Coumaric Acid	Caftaric Acid	Coutaric Acid	Caffeic Acid	(+)-Catechin	(−)-Epicatechin	Malvidin-3-Glucoside	Delphinidin-3-Glucoside
Me21	9.53 ± 0.06 ^j^	13.31 ± 0.14 ^f^	26.85 ± 0.01 ^c^	7.32 ± 0.06 ^f^	27.06 ± 0.33 ^f^	9.21 ± 0.09 ^e^	23.81 ± 0.26 ^i^	80.52 ± 0.45 ^f^	31.93 ± 0.26 ^i^	12.71 ± 0.01 ^d^	4.00 ± 0.06 ^f^
1A	8.78 ± 0.02 ^ef^	12.38 ± 0.01 ^b^	24.18 ± 0.12 ^a^	6.55 ± 0.04 ^d^	24.26 ± 0.17 ^e^	8.30 ± 0.07 ^d^	21.35 ± 0.15 ^g^	77.60 ± 0.41 ^d^	28.50 ± 0.28 ^f^	11.75 ± 0.05 ^c^	3.68 ± 0.20 ^de^
2A	8.92 ± 0.01 ^g^	12.60 ± 0.02 ^c^	31.33 ± 0.01 ^i^	4.74 ± 0.01 ^a^	41.07 ± 0.01 ^j^	12.57 ± 0.01 ^i^	13.15 ± 0.01 ^b^	78.58 ± 0.15 ^e^	27.26 ± 0.01 ^de^	11.60 ± 0.02 ^c^	3.65 ± 0.01 ^de^
3A	9.71 ± 0.08 ^k^	13.19 ± 0.11 ^e^	32.40 ± 0.05 ^j^	4.90 ± 0.02 ^a^	41.27 ± 0.05 ^j^	12.63 ± 0.05 ^i^	13.49 ± 0.17 ^b^	79.96 ± 0.01 ^f^	28.04 ± 0.15 ^f^	11.12 ± 0.07 ^c^	3.61 ± 0.01 ^de^
4A	10.78 ± 0.01 ^m^	16.01 ± 0.06 ^j^	33.71 ± 0.25 ^k^	5.73 ± 0.04 ^b^	45.29 ± 0.41 ^k^	13.70 ± 0.14 ^j^	15.30 ± 0.09 ^c^	85.37 ± 0.70 ^hi^	37.94 ± 0.01 ^j^	10.37 ± 0.21 ^b^	3.45 ± 0.02 ^d^
1B	9.02 ± 0.02 ^h^	13.00 ± 0.01 ^e^	31.41 ± 0.17 ^i^	5.74 ± 0.02 ^b^	35.76 ± 0.13 ^i^	11.24 ± 0.04 ^g^	16.85 ± 0.06 ^d^	78.31 ± 0.06 ^e^	28.63 ± 0.12 ^f^	13.51 ± 0.16 ^e^	3.74 ± 0.03 ^e^
2B	8.29 ± 0.01 ^b^	12.64 ± 0.07 ^c^	27.52 ± 0.14 ^d^	6.82 ± 0.03 ^d^	21.85 ± 0.11 ^b^	7.19 ± 0.24 ^b^	22.30 ± 0.09 ^h^	77.13 ± 0.14 ^cd^	27.42 ± 0.02 ^e^	12.87 ± 0.02 ^d^	3.76 ± 0.02 ^e^
3B	9.27 ± 0.08 ^i^	13.77 ± 0.01 ^g^	29.95 ± 0.25 ^f^	7.86 ± 0.01 ^h^	21.29 ± 0.05 ^b^	6.60 ± 0.09 ^a^	26.23 ± 0.04 ^k^	80.38 ± 0.18 ^f^	30.46 ± 0.27 ^h^	12.39 ± 0.03 ^d^	3.72 ± 0.02 ^e^
4B	9.98 ± 0.02 ^l^	15.17 ± 0.03 ^i^	30.68 ± 0.09 ^h^	7.94 ± 0.05 ^h^	23.41 ± 0.02 ^d^	7.98 ± 0.11 ^cd^	25.92 ± 0.23 ^k^	86.21 ± 0.07 ^i^	30.83 ± 0.03 ^h^	11.23 ± 0.03 ^c^	3.47 ± 0.02 ^d^
1C	8.27 ± 0.01 ^b^	12.12 ± 0.03 ^a^	25.25 ± 0.08 ^b^	6.02 ± 0.03 ^c^	23.84 ± 0.08 ^d^	8.15 ± 0.06 ^d^	19.09 ± 0.06 ^f^	73.28 ± 0.02 ^a^	24.88 ± 0.11 ^b^	11.87 ± 0.12 ^c^	3.21 ± 0.06 ^c^
2C	7.85 ± 0.10 ^a^	12.19 ± 0.05 ^a^	28.13 ± 0.17 ^e^	6.77 ± 0.06 ^d^	23.04 ± 0.11 ^d^	7.97 ± 0.06 ^c^	22.22 ± 0.07 ^h^	76.83 ± 0.39 ^c^	27.64 ± 0.17 ^e^	12.44 ± 0.06 ^d^	3.69 ± 0.11 ^de^
3C	8.40 ± 0.02 ^d^	12.37 ± 0.08 ^b^	27.30 ± 0.15 ^d^	6.77 ± 0.04 ^d^	19.69 ± 0.06 ^a^	6.17 ± 0.06 ^a^	22.75 ± 0.10 ^h^	75.03 ± 0.20 ^ab^	27.27 ± 0.04 ^de^	12.70 ± 0.05 ^d^	3.50 ± 0.02 ^d^
4C	8.69 ± 0.05 ^e^	14.81 ± 0.04 ^h^	30.91 ± 0.14 ^h^	7.96 ± 0.06 ^h^	21.53 ± 0.06 ^b^	7.77 ± 0.03 ^c^	26.18 ± 0.08 ^k^	86.52 ± 0.80 ^i^	30.56 ± 0.32 ^h^	11.36 ± 0.29 ^c^	3.27 ± 0.11 ^c^
1D	8.84 ± 0.01 ^f^	12.81 ± 0.02 ^d^	31.31 ± 0.20 ^i^	5.81 ± 0.01 ^b^	35.23 ± 0.42 ^i^	11.06 ± 0.11 ^g^	17.08 ± 0.13 ^d^	77.44 ± 0.38 ^cd^	26.85 ± 0.30 ^d^	11.38 ± 0.02 ^c^	3.13 ± 0.04 ^c^
2D	8.35 ± 0.05 ^d^	12.79 ± 0.04 ^cd^	28.36 ± 0.09 ^e^	7.05 ± 0.01 ^e^	22.65 ± 0.01 ^c^	6.98 ± 0.21 ^b^	22.71 ± 0.01 ^h^	71.36 ± 0.10 ^b^	24.57 ± 0.02 ^b^	11.40 ± 0.08 ^c^	2.82 ± 0.03 ^b^
3D	8.21 ± 0.05 ^b^	13.29 ± 0.01 ^f^	30.37 ± 0.06 ^g^	7.62 ± 0.03 ^g^	21.17 ± 0.05 ^b^	7.47 ± 0.01 ^b^	24.55 ± 0.05 ^j^	78.14 ± 0.30 ^de^	24.07 ± 0.03 ^b^	11.06 ± 0.02 ^c^	2.88 ± 0.01 ^b^
4D	8.87 ± 0.01 ^f^	15.12 ± 0.11 ^i^	31.07 ± 0.16 ^hi^	7.92 ± 0.04 ^h^	22.00 ± 0.56 ^bc^	7.76 ± 0.02 ^c^	26.04 ± 0.09 ^k^	86.63 ± 0.45 ^i^	29.93 ± 0.18 ^g^	10.50 ± 0.16 ^b^	2.74 ± 0.10 ^b^
1E	7.94 ± 0.05 ^a^	12.48 ± 0.01 ^b^	30.27 ± 0.03 ^g^	4.64 ± 0.01 ^a^	40.51 ± 0.57 ^j^	12.20 ± 0.02 ^h^	11.48 ± 0.01 ^a^	74.04 ± 0.32 ^a^	22.93 ± 0.26 ^a^	10.84 ± 0.01 ^b^	2.82 ± 0.01 ^b^
2E	8.57 ± 0.01 ^e^	13.02 ± 0.01 ^e^	29.51 ± 0.02 ^f^	5.68 ± 0.04 ^b^	33.39 ± 0.01 ^h^	10.28 ± 0.41 ^f^	17.14 ± 0.01 ^d^	76.47 ± 0.52 ^c^	27.36 ± 0.17 ^de^	11.17 ± 0.08 ^c^	3.10 ± 0.03 ^c^
3E	8.17 ± 0.01 ^b^	12.39 ± 0.03 ^b^	27.08 ± 0.08 ^d^	5.68 ± 0.01 ^b^	27.72 ± 0.20 ^f^	8.20 ± 0.02 ^d^	17.66 ± 0.01 ^e^	74.41 ± 0.29 ^a^	25.62 ± 0.13 ^c^	10.69 ± 0.06 ^b^	2.44 ± 0.01 ^a^
4E	8.05 ± 0.04 ^a^	14.61 ± 0.02 ^g^	30.92 ± 0.09 ^h^	6.81 ± 0.01 ^d^	29.34 ± 0.06 ^g^	9.68 ± 0.03 ^e^	21.36 ± 0.08 ^g^	83.61 ± 0.10 ^g^	29.84 ± 0.12 ^g^	8.55 ± 0.01 ^a^	2.40 ± 0.01 ^a^

Significant differences (*p* < 0.05) in the same column have been marked with different superscript letters (a–m). Me21—Merlot vintage 2021 sample before ageing; A—stainless-steel tank; B—Excellence wooden barrel with medium toasting; C—Excellence wooden barrel with medium-plus toasting; D—Excellence wooden barrel with medium-long toasting; E—Premium wooden barrel with medium toasting; 1A–1E—sampling in August 2022; 2A–2E—sampling in November 2022; 3A–3E—sampling in February 2023; 4A–4E—sampling in May 2023.

The concentrations of individual phenolic compounds varied among samples, regarding vessel type, wine vintage, and ageing time. Regarding the 2020 vintage Merlot, after 12 months of ageing, the highest concentrations of almost all of the analysed phenolic compounds (except p-coumaric and caffeic acid) were measured in the SST. The initial concentrations of quercetin (10.60 mg/L), hyperoside (10.22 mg/L), and coutaric acid (15.34 mg/L) increased after ageing in SST, and the concentrations of the gallic (28.33 mg/L) and caftaric acid (46.68 mg/L), (+)-catechin (58.36 mg/L), (−)-epicatechin (19.34 mg/L), malvidin-3-glucoside (10.51 mg/L), and delphinidin-3-glucoside (2.68 mg/L) decreased. However, the wooden barrels with different toasting levels were less favourable for the mentioned compounds than the SST, especially EMT for quercetin, hyperoside, and caftaric and coutaric acid; EMT+ for (+)-catechin and the two anthocyanins; PMT also for delphinidin-3-glucoside, where the lowest concentrations were measured after 12 months of ageing. For (−)-epicatechin, the lowest concentration was consistently measured in all wooden barrels after 12 months of ageing, with no significant differences between them (the average concentration was 13.04 mg/L).

The SST was the least favourable only for p-coumaric and caffeic acid, where the lowest concentrations were recorded after 12 months of ageing in Me20 (7.75 mg/L and 10.57 mg/L, respectively). However, these concentrations were still slightly higher than the initial values (6.44 mg/L and 8.09 mg/L, respectively). The highest concentrations of caffeic acid were found in the EMT and EMLT barrels (23.54 mg/L and 23.20 mg/L, respectively), while p-coumaric acid concentrations were highest in all wooden barrels, with no significant difference observed between them.

Regarding the 2021 Merlot, there were some similarities in the behaviour of individual phenolic compounds during ageing in different vessels. For example, the SST resulted in the lowest concentrations of p-coumaric acid (5.73 mg/L) and caffeic acid (15.30 mg/L), while all Excellence wooden barrels with different toasting methods resulted in an 8.5–9.5% increase in initial concentrations, with no significant difference among samples. The initial concentrations of malvidin-3-glucoside (12.71 mg/L) and delphinidin-3-glucoside (4.00 mg/L) decreased after ageing in all vessels, especially in the PMT after 12 months of ageing (8.55 and 2.40 mg/L, respectively). The SST was most favourable for quercetin, hyperoside, gallic, caftaric and coutaric acid, and (−)-epicatechin, where the highest concentrations were measured after 12 months of ageing. Although the initial concentration of (+)-catechin (80.52 mg/L) increased after ageing in SST, the Excellence wooden barrels with different toasting methods were more favourable for this compound, where its concentration increased above 86 mg/L after 12 months of ageing. The PMT barrel resulted in the lowest concentrations of quercetin (8.05 mg/L) and hyperoside (14.61 mg/L) after 12 months of ageing of 2021 Merlot in it. Unlike in the Me20 samples, the initial concentrations of gallic acid (26.85 mg/L) increased after 12 months of ageing in all vessels, especially in the SST (33.71 mg/L).

To compare individual phenolic compounds between vessels and the two wine vintages, a PCA biplot was generated (Figure 1). 

Principal Component 1 (PC1), which explained 67.78% of the total variance, effectively separated the 2020 and 2021 vintage Merlot samples, with the 2021 vintage positioned on the positive side, grouping most of the phenolic compounds. The exception was p-coumaric acid, which clustered with the 2020 vintage Merlot after ageing in wooden barrels with varying toasting methods for 9 and 12 months. In addition to the clear distinction between the phenolic profiles of the two vintages, noticeable differences were also observed after ageing in the SST and the four wooden barrels. Samples from the SST were placed near the corresponding initial wine, positioned on the negative side of Principal Component 2 (PC2), which accounted for 27.01% of the total variance. Coutaric acid, caftaric acid, and quercetin were associated with the 2021 vintage Merlot aged in SST, regardless of the ageing time. Wooden barrels and extended ageing times induced more significant changes in the phenolic profiles of both vintages compared to the initial wines. Although some slight variations were found among the samples aged in wooden barrels, they all clustered together. Gallic acid, hyperoside, the two flavan-3-ols, and the two anthocyanins were grouped with the 2021 vintage Merlot aged in wooden barrels with different toasting levels.

### 3.3. CIELab Colour Parameters

The CIELab colour parameters of the analysed wines were measured to assess the colour changes during ageing in different vessels. The results, shown in Table 8 and Table 9, indicate that both wine vintages exhibited similar initial L* and b* values (20.06 and 0.59, respectively). However, the initial a* (0.53) and C* (0.80) values were slightly higher for the 2021 vintage Merlot (Me21) compared to the 2020 vintage (Me20), which had a* and C* values of 0.35 and 0.68, respectively. The hue angle (°h) was slightly lower for Me20 (59.28°) than for Me21 (47.97°).

**Table 8:** CIELab parameters of 2020 vintage Merlot and samples obtained after 12 months of ageing in different vessels.

Sample	L*	a*	b*	°h	C*	ΔE*
Me20	20.06 ± 0.01 ^f^	0.35 ± 0.01 ^e^	0.59 ± 0.01 ^c^	59.28 ± 0.10 ^k^	0.68 ± 0.01 ^c^	
1a	19.95 ± 0.01 ^e^	0.42 ± 0.02 ^f^	0.58 ± 0.01 ^c^	54.52 ± 0.54 ^h^	0.71 ± 0.01 ^cd^	0.09 ± 0.01 ^a^
2a	19.96 ± 0.02 ^e^	0.30 ± 0.03 ^d^	0.60 ± 0.01 ^c^	63.37 ± 0.71 ^m^	0.67 ± 0.01 ^c^	0.07 ± 0.01 ^a^
3a	19.88 ± 0.01 ^de^	0.11 ± 0.01 ^a^	0.52 ± 0.02 ^b^	77.68 ± 0.13 ^o^	0.54 ± 0.02 ^a^	0.28 ± 0.01 ^c^
4a	20.17 ± 0.02 ^gh^	0.24 ± 0.02 ^c^	0.44 ± 0.01 ^a^	61.34 ± 0.25 ^l^	0.50 ± 0.01 ^a^	0.25 ± 0.01 ^c^
1b	20.20 ± 0.02 ^h^	0.60 ± 0.03 ^h^	0.57 ± 0.01 ^c^	43.49 ± 0.08 ^c^	0.83 ± 0.02 ^ef^	0.32 ± 0.01 ^cd^
2b	20.13 ± 0.01 ^g^	0.72 ± 0.02 ^j^	0.61 ± 0.02 ^c^	40.39 ± 0.05 ^b^	0.94 ± 0.03 ^g^	0.39 ± 0.01 ^de^
3b	19.97 ± 0.01 ^e^	0.49 ± 0.02 ^g^	0.60 ± 0.01 ^c^	51.14 ± 0.37 ^f^	0.78 ± 0.01 ^e^	0.15 ± 0.01 ^b^
4b	19.92 ± 0.01 ^e^	0.82 ± 0.02 ^k^	0.64 ± 0.03 ^cd^	37.87 ± 0.50 ^a^	1.05 ± 0.02 ^h^	0.48 ± 0.01 ^f^
1c	19.81 ± 0.02 ^d^	0.44 ± 0.01 ^f^	0.66 ± 0.02 ^cd^	56.48 ± 0.75 ^j^	0.79 ± 0.02 ^e^	0.23 ± 0.01 ^c^
2c	19.67 ± 0.03 ^c^	0.47 ± 0.01 ^g^	0.62 ± 0.01 ^c^	53.80 ± 0.90 ^h^	0.79 ± 0.01 ^e^	0.36 ± 0.01 ^d^
3c	19.70 ± 0.02 ^c^	0.40 ± 0.01 ^f^	0.61 ± 0.02 ^c^	56.52 ± 0.28 ^j^	0.73 ± 0.01 ^d^	0.31 ± 0.01 ^cd^
4c	19.65 ± 0.02 ^c^	0.18 ± 0.01 ^b^	0.59 ± 0.02 ^c^	73.50 ± 0.35 ^n^	0.61 ± 0.02 ^b^	0.40 ± 0.01 ^d^
1d	19.64 ± 0.01 ^bc^	0.55 ± 0.02 ^gh^	0.69 ± 0.02 ^d^	51.25 ± 0.34 ^fg^	0.88 ± 0.02 ^fg^	0.43 ± 0.01 ^e^
2d	19.62 ± 0.01 ^bc^	0.56 ± 0.02 ^h^	0.73 ± 0.02 ^e^	52.78 ± 0.63 ^g^	0.92 ± 0.01 ^g^	0.47 ± 0.01 ^f^
3d	19.68 ± 0.03 ^c^	0.57 ± 0.01 ^h^	0.74 ± 0.01 ^e^	52.08 ± 0.34 ^fg^	0.93 ± 0.02 ^g^	0.42 ± 0.01 ^e^
4d	19.71 ± 0.02 ^c^	0.67 ± 0.01 ^i^	0.77 ± 0.02 ^e^	49.03 ± 0.57 ^e^	1.03 ± 0.01 ^h^	0.48 ± 0.01 ^f^
1e	19.56 ± 0.02 ^b^	0.49 ± 0.02 ^g^	0.71 ± 0.04 ^de^	55.56 ± 0.08 ^i^	0.87 ± 0.01 ^f^	0.49 ± 0.01 ^f^
2e	19.49 ± 0.01 ^a^	0.68 ± 0.01 ^i^	0.75 ± 0.02 ^e^	47.87 ± 0.60 ^d^	1.02 ± 0.01 ^h^	0.63 ± 0.01 ^g^
3e	19.58 ± 0.02 ^b^	0.40 ± 0.02 ^f^	0.69 ± 0.01 ^d^	59.89 ± 0.49 ^k^	0.80 ± 0.01 ^e^	0.44 ± 0.01 ^ef^
4e	19.70 ± 0.01 ^c^	0.53 ± 0.01 ^g^	0.68 ± 0.01 ^d^	52.05 ± 0.34 ^fg^	0.86 ± 0.01 ^f^	0.37 ± 0.01 ^d^

Significant differences (*p* < 0.05) in the same column have been marked with different superscript letters (a–o). Me20—Merlot vintage 2020 sample before ageing; a—stainless-steel tank; b—Excellence wooden barrel with medium toasting; c—Excellence wooden barrel with medium-plus toasting; d—Excellence wooden barrel with medium-long toasting; e—Premium wooden barrel with medium toasting; 1a–1e—sampling in June 2021; 2a–2e—sampling in September 2021; 3a–3e—sampling in December 2021; 4a–4e—sampling in March 2022.

**Table 9:** CIELab parameters of 2021 vintage Merlot and samples obtained after 12 months of ageing in different vessels.

Sample	L*	a*	b*	°h	C*	ΔE*
Me21	20.06 ± 0.01 ^ef^	0.53 ± 0.01 ^f^	0.59 ± 0.01 ^a^	47.97 ± 0.64 ^f^	0.80 ± 0.01 ^c^	
1A	20.09 ± 0.02 ^f^	0.21 ± 0.01 ^b^	0.57 ± 0.01 ^a^	69.36 ± 0.55 ^kl^	0.61 ± 0.01 ^a^	0.32 ± 0.01 ^d^
2A	20.04 ± 0.02 ^e^	0.35 ± 0.01 ^e^	0.57 ± 0.01 ^a^	58.19 ± 0.70 ^hi^	0.67 ± 0.01 ^b^	0.18 ± 0.01 ^a^
3A	20.02 ± 0.02 ^e^	0.36 ± 0.01 ^e^	0.55 ± 0.01 ^a^	57.03 ± 0.88 ^h^	0.66 ± 0.01 ^b^	0.18 ± 0.01 ^a^
4A	19.54 ± 0.01 ^a^	0.19 ± 0.01 ^ab^	0.79 ± 0.01 ^cd^	76.17 ± 0.42 ^o^	0.82 ± 0.01 ^c^	0.65 ± 0.01 ^h^
1B	20.02 ± 0.01 ^e^	0.16 ± 0.01 ^a^	0.55 ± 0.01 ^a^	73.41 ± 0.74 ^n^	0.57 ± 0.01 ^a^	0.37 ± 0.01 ^e^
2B	19.63 ± 0.02 ^b^	0.70 ± 0.02 ^h^	0.70 ± 0.01 ^bc^	44.79 ± 0.14 ^e^	1.00 ± 0.02 ^f^	0.48 ± 0.01 ^f^
3B	19.85 ± 0.03 ^d^	0.84 ± 0.02 ^i^	0.67 ± 0.01 ^b^	38.59 ± 0.39 ^bc^	1.08 ± 0.01 ^g^	0.39 ± 0.01 ^e^
4B	19.50 ± 0.01 ^a^	0.34 ± 0.01 ^de^	0.83 ± 0.02 ^d^	67.63 ± 0.39 ^h^	0.90 ± 0.01 ^e^	0.64 ± 0.01 ^h^
1C	19.87 ± 0.02 ^d^	0.31 ± 0.01 ^d^	0.60 ± 0.01 ^ab^	62.72 ± 0.66 ^j^	0.67 ± 0.02 ^b^	0.29 ± 0.01 ^cd^
2C	19.79 ± 0.01 ^c^	0.65 ± 0.02 ^g^	0.61 ± 0.01 ^ab^	43.46 ± 0.96 ^de^	0.89 ± 0.01 ^de^	0.30 ± 0.01 ^d^
3C	19.88 ± 0.01 ^d^	0.72 ± 0.02 ^h^	0.67 ± 0.01 ^b^	43.02 ± 0.44 ^d^	0.98 ± 0.02 ^f^	0.27 ± 0.01 ^c^
4C	19.55 ± 0.01 ^a^	0.50 ± 0.01 ^f^	0.84 ± 0.02 ^d^	59.65 ± 1.06 ^i^	0.98 ± 0.02 ^f^	0.57 ± 0.01 ^g^
1D	19.83 ± 0.02 ^cd^	0.34 ± 0.01 ^de^	0.56 ± 0.01 ^a^	58.66 ± 0.96 ^hi^	0.66 ± 0.01 ^b^	0.30 ± 0.01 ^d^
2D	19.87 ± 0.03 ^d^	0.66 ± 0.01 ^g^	0.60 ± 0.01 ^ab^	42.24 ± 0.90 ^d^	0.89 ± 0.02 ^de^	0.23 ± 0.01 ^b^
3D	19.73 ± 0.01 ^c^	0.89 ± 0.02 ^ij^	0.74 ± 0.01 ^c^	39.95 ± 0.85 ^c^	1.15 ± 0.02 ^g^	0.51 ± 0.01 ^f^
4D	19.52 ± 0.01 ^a^	0.24 ± 0.01 ^bc^	0.82 ± 0.02 ^d^	73.65 ± 0.12 ^m^	0.86 ± 0.01 ^d^	0.65 ± 0.01 ^h^
1E	19.89 ± 0.01 ^d^	0.91 ± 0.02 ^j^	0.69 ± 0.01 ^b^	37.55 ± 0.06 ^b^	1.14 ± 0.03 ^g^	0.42 ± 0.01 ^ef^
2E	19.76 ± 0.01 ^c^	0.46 ± 0.01 ^f^	0.66 ± 0.01 ^b^	55.02 ± 0.87 ^g^	0.81 ± 0.01 ^c^	0.31 ± 0.01 ^d^
3E	19.72 ± 0.03 ^c^	1.06 ± 0.02 ^k^	0.76 ± 0.01 ^c^	35.55 ± 0.11 ^a^	1.30 ± 0.02 ^h^	0.65 ± 0.01 ^h^
4E	19.53 ± 0.02 ^a^	0.26 ± 0.01 ^c^	0.78 ± 0.01 ^c^	71.60 ± 0.44 ^l^	0.83 ± 0.01 ^c^	0.63 ± 0.01 ^h^

Significant differences (*p* < 0.05) in the same column have been marked with different superscript letters (a–o). Me21—Merlot vintage 2021 sample before ageing; A—stainless-steel tank; B—Excellence wooden barrel with medium toasting; C—Excellence wooden barrel with medium-plus toasting; D—Excellence wooden barrel with medium-long toasting; E—Premium wooden barrel with medium toasting; 1A–1E—sampling in August 2022; 2A–2E—sampling in November 2022; 3A–3E—sampling in February 2023; 4A–4E—sampling in May 2023.

During ageing, the changes in these parameters varied between the vessels. The lightness (L*) decreased after 12 months of ageing in all vessels for both vintages, except for Me20 aged in the SST, where L* increased slightly to 20.17. A similar trend was observed for the a* value in Me21, which decreased after ageing in all vessels, except for the EMT+ barrel, where there was no significant change from the initial value. The b* value increased after 12 months in all vessels for both wines, except for Me20 aged in SST, where it decreased to 0.44.

The hue angle (°h) increased after 12 months of ageing in all vessels for Me21, with a notable increase in SST (76.17°). For Me20, the hue angle increased only in SST and EMT+ after 12 months, reaching 61.34° and 73.50°, respectively. The highest C* value among the Me20 samples was observed in the EMT and EMLT barrels (1.05 and 1.03, respectively), while the highest C* value for Me21 was found in the EMT+ barrel (0.98).

The ΔE* values, which indicate the colour change relative to the initial wine, were below 1 in all samples. The highest ΔE* value for Me20 was measured after 12 months in the EMT and EMLT barrels (0.48 for both). For Me21, ΔE* increased during ageing in all vessels, reaching an average of 0.64, except in the EMT+ barrel, where the lowest value was recorded (0.57).

### 3.4. Element Content

In this study, concentrations of 20 elements (boron, sodium, aluminium, calcium, vanadium, chromium, manganese, iron, cobalt, nickel, copper, zinc, arsenic, selenium, strontium, molybdenum, tin, antimony, barium, and led) were measured, and the results were presented in Table 10 and Table 11.

**Table 10:** Element content in Merlot vintage 2020 and samples obtained during 12-month ageing in different vessels.

Sample	B (mg/L)	Na (mg/L)	Al (μg/L)	Ca (mg/L)	V (μg/L)	Cr (μg/L)	Mn (μg/L)	Fe (μg/L)	Co (μg/L)	Ni (μg/L)
Me20	3.50 ± 0.05 ^b^	4.06 ± 0.05 ^c^	146.89 ± 1.12 ^c^	5.62 ± 0.32 ^ab^	0.43 ± 0.05 ^d^	4.64 ± 0.51 ^de^	598.84 ± 13.75 ^ab^	874.73 ± 22.84 ^g^	3.13 ± 0.09 ^bc^	14.92 ± 0.68 ^f^
1a	3.55 ± 0.13 ^ab^	3.48 ± 0.07 ^a^	145.46 ± 5.63 ^c^	6.63 ± 0.02 ^c^	0.29 ± 0.06 ^ab^	6.06 ± 0.63 ^ef^	576.85 ± 11.63 ^ab^	654.29 ± 14.19 ^cd^	3.23 ± 0.08 ^bc^	10.06 ± 0.63 ^bc^
2a	3.45 ± 0.02 ^b^	3.67 ± 0.07 ^b^	135.51 ± 10.47 ^bc^	5.86 ± 0.15 ^b^	0.34 ± 0.02 ^c^	4.81 ± 0.76 ^de^	577.11 ± 7.32 ^ab^	568.95 ± 12.67 ^a^	3.08 ± 0.05 ^b^	10.26 ± 0.48 ^bc^
3a	3.53 ± 0.08 ^b^	3.67 ± 0.04 ^b^	155.51 ± 3.56 ^d^	5.76 ± 0.29 ^ab^	0.41 ± 0.08 ^cd^	8.78 ± 0.56 ^g^	582.66 ± 16.07 ^ab^	686.83 ± 8.19 ^d^	3.31 ± 0.08 ^c^	11.75 ± 0.05 ^d^
4a	3.52 ± 0.12 ^b^	3.68 ± 0.08 ^b^	121.64 ± 2.04 ^b^	5.44 ± 0.13 ^a^	0.36 ± 0.04 ^c^	3.78 ± 0.04 ^c^	569.06 ± 5.78 ^ab^	677.49 ± 33.15 ^cd^	3.08 ± 0.02 ^b^	10.33 ± 0.29 ^c^
1b	3.51 ± 0.10 ^b^	3.80 ± 0.19 ^bc^	137.56 ± 12.13 ^bc^	6.71 ± 0.37 ^cd^	0.27 ± 0.02 ^ab^	5.12 ± 0.40 ^de^	602.94 ± 13.56 ^b^	760.32 ± 27.49 ^ef^	2.90 ± 0.08 ^a^	9.85 ± 0.01 ^b^
2b	3.30 ± 0.03 ^a^	3.76 ± 0.01 ^b^	136.52 ± 10.18 ^bc^	6.98 ± 0.24 ^cd^	0.22 ± 0.03 ^a^	2.50 ± 0.24 ^a^	611.06 ± 3.06 ^b^	773.08 ± 0.09 ^f^	3.06 ± 0.03 ^b^	9.69 ± 0.04 ^b^
3b	3.30 ± 0.06 ^a^	3.84 ± 0.09 ^bc^	127.17 ± 9.18 ^ab^	6.40 ± 0.22 ^c^	0.23 ± 0.03 ^ab^	6.33 ± 0.81 ^ef^	603.89 ± 17.52 ^bc^	615.13 ± 25.71 ^bc^	3.01 ± 0.08 ^ab^	12.94 ± 0.06 ^e^
4b	3.27 ± 0.02 ^a^	3.92 ± 0.11 ^c^	139.65 ± 9.22 ^b^	6.24 ± 0.27 ^bc^	0.28 ± 0.01 ^b^	2.82 ± 0.37 ^ab^	632.57 ± 13.68 ^c^	644.19 ± 19.17 ^c^	3.21 ± 0.03 ^bc^	11.01 ± 0.06 ^d^
1c	3.35 ± 0.05 ^ab^	3.79 ± 0.13 ^bc^	143.31 ± 10.13 ^bcd^	6.27 ± 0.08 ^c^	0.22 ± 0.03 ^a^	3.01 ± 0.14 ^ab^	579.39 ± 14.27 ^ab^	737.92 ± 15.70 ^ef^	3.02 ± 0.08 ^ab^	8.89 ± 0.08 ^a^
2c	3.46 ± 0.21 ^ab^	4.10 ± 0.15 ^cd^	148.07 ± 2.68 ^c^	6.87 ± 0.33 ^c^	0.29 ± 0.03 ^b^	5.34 ± 0.96 ^de^	620.52 ± 25.46 ^bc^	669.19 ± 31.66 ^cd^	3.24 ± 0.01 ^c^	11.58 ± 0.08 ^d^
3c	3.31 ± 0.09 ^a^	4.03 ± 0.05 ^c^	143.94 ± 8.91 ^bc^	6.23 ± 0.09 ^c^	0.20 ± 0.02 ^a^	5.35 ± 0.25 ^e^	608.17 ± 15.09 ^bc^	678.16 ± 12.87 ^d^	3.12 ± 0.09 ^bc^	12.72 ± 0.07 ^e^
4c	3.28 ± 0.09 ^a^	3.95 ± 0.13 ^c^	115.22 ± 2.80 ^a^	5.84 ± 0.16 ^b^	0.18 ± 0.03 ^a^	5.11 ± 0.24 ^de^	595.52 ± 11.95 ^b^	631.94 ± 13.20 ^bc^	2.99 ± 0.10 ^ab^	11.47 ± 0.09 ^d^
1d	3.39 ± 0.11 ^ab^	3.85 ± 0.19 ^bc^	131.83 ± 10.31 ^bc^	6.34 ± 0.23 ^c^	0.34 ± 0.02 ^c^	4.72 ± 0.47 ^de^	590.22 ± 25.11 ^ab^	752.44 ± 29.68 ^ef^	2.96 ± 0.12 ^ab^	10.22 ± 0.06 ^c^
2d	3.34 ± 0.13 ^ab^	4.30 ± 0.20 ^cd^	132.21 ± 5.32 ^b^	7.20 ± 0.28 ^d^	0.24 ± 0.01 ^a^	4.71 ± 0.17 ^d^	606.92 ± 19.67 ^bc^	652.17 ± 20.17 ^cd^	3.06 ± 0.02 ^b^	10.66 ± 0.05 ^c^
3d	3.33 ± 0.08 ^ab^	4.35 ± 0.06 ^d^	134.11 ± 2.17 ^b^	6.47 ± 0.13 ^c^	0.21 ± 0.04 ^a^	6.67 ± 0.17 ^f^	605.89 ± 4.65 ^b^	647.97 ± 4.02 ^c^	3.08 ± 0.08 ^b^	10.13 ± 0.26 ^c^
4d	3.22 ± 0.05 ^a^	4.17 ± 0.08 ^cd^	126.84 ± 2.22 ^ab^	6.01 ± 0.12 ^bc^	0.19 ± 0.04 ^a^	4.56 ± 0.17 ^d^	603.70 ± 3.55 ^b^	610.84 ± 8.05 ^b^	2.95 ± 0.01 ^a^	10.36 ± 0.04 ^c^
1e	3.36 ± 0.13 ^ab^	3.67 ± 0.10 ^ab^	139.26 ± 6.69 ^bc^	7.81 ± 0.26 ^d^	0.19 ± 0.02 ^a^	3.42 ± 0.10 ^b^	615.28 ± 8.39 ^bc^	786.30 ± 25.06 ^f^	3.06 ± 0.07 ^b^	10.28 ± 0.08 ^c^
2e	3.38 ± 0.12 ^ab^	3.79 ± 0.05 ^bc^	131.99 ± 1.33 ^b^	6.26 ± 0.17 ^c^	0.21 ± 0.01 ^a^	2.30 ± 0.16 ^a^	629.40 ± 5.33 ^c^	747.22 ± 20.87 ^ef^	3.12 ± 0.05 ^b^	12.24 ± 0.18 ^e^
3e	3.45 ± 0.12 ^ab^	3.91 ± 0.11 ^c^	133.78 ± 2.50 ^b^	5.91 ± 0.23 ^b^	0.20 ± 0.04 ^a^	3.45 ± 0.23 ^b^	648.99 ± 20.96 ^cd^	724.77 ± 24.70 ^ef^	3.25 ± 0.03 ^c^	11.54 ± 0.11 ^d^
4e	3.46 ± 0.06 ^ab^	4.22 ± 0.05 ^cd^	134.25 ± 1.70 ^b^	5.63 ± 0.07 ^ab^	0.21 ± 0.03 ^a^	3.10 ± 0.19 ^b^	654.37 ± 8.45 ^d^	721.62 ± 7.47 ^e^	3.14 ± 0.04 ^b^	11.80 ± 0.06 ^d^
Sample	Cu (μg/L)	Zn (μg/L)	As (μg/L)	Se (μg/L)	Sr (μg/L)	Mo (μg/L)	Sn (μg/L)	Sb (μg/L)	Ba (μg/L)	Pb (μg/L)
Me20	186.26 ± 2.59 ^m^	394.56 ± 3.42 ^e^	3.87 ± 0.12 ^a^	4.46 ± 0.33 ^d^	780.52 ± 12.99 ^ab^	0.60 ± 0.03 ^b^	1.63 ± 0.17 ^d^	0.16 ± 0.03 ^ab^	38.60 ± 0.50 ^a^	2.02 ± 0.28 ^ab^
1a	74.20 ± 0.93 ^j^	360.54 ± 6.03 ^c^	5.44 ± 0.47 ^c^	4.05 ± 0.27 ^d^	745.62 ± 13.53 ^a^	0.42 ± 0.01 ^a^	1.60 ± 0.24 ^d^	0.56 ± 0.10 ^d^	43.25 ± 0.38 ^b^	2.21 ± 0.26 ^b^
2a	64.24 ± 1.42 ^i^	356.49 ± 5.32 ^c^	5.45 ± 0.15 ^c^	4.63 ± 0.48 ^d^	738.37 ± 20.52 ^a^	0.46 ± 0.04 ^a^	0.24 ± 0.02 ^a^	0.45 ± 0.12 ^d^	43.20 ± 0.29 ^b^	1.77 ± 0.21 ^a^
3a	31.76 ± 1.65 ^g^	376.56 ± 1.41 ^d^	5.25 ± 0.63 ^c^	4.09 ± 0.14 ^d^	745.07 ± 8.51 ^a^	0.75 ± 0.09 ^c^	0.35 ± 0.05 ^b^	0.27 ± 0.02 ^c^	44.37 ± 0.74 ^c^	1.77 ± 0.12 ^a^
4a	16.94 ± 0.34 ^a^	364.51 ± 3.56 ^c^	4.61 ± 0.61 ^b^	4.61 ± 0.89 ^d^	745.44 ± 5.43 ^a^	0.75 ± 0.05 ^c^	0.34 ± 0.04 ^b^	0.26 ± 0.08 ^bc^	41.81 ± 0.64 ^b^	1.60 ± 0.23 ^a^
1b	102.10 ± 2.38 ^kl^	339.09 ± 7.02 ^ab^	4.14 ± 0.35 ^ab^	2.26 ± 0.37 ^c^	809.67 ± 10.89 ^b^	0.81 ± 0.01 ^c^	1.33 ± 0.26 ^d^	0.24 ± 0.04 ^bc^	57.35 ± 0.35 ^e^	3.61 ± 0.24 ^d^
2b	111.35 ± 0.48 ^l^	380.66 ± 2.28 ^d^	3.87 ± 0.05 ^a^	2.62 ± 0.29 ^c^	798.52 ± 6.80 ^ab^	0.80 ± 0.07 ^c^	0.43 ± 0.02 ^c^	0.17 ± 0.02 ^ab^	63.03 ± 1.08 ^f^	4.13 ± 0.62 ^de^
3b	19.17 ± 0.76 ^b^	334.12 ± 1.61 ^a^	3.26 ± 0.46 ^a^	1.76 ± 0.20 ^b^	791.99 ± 29.87 ^ab^	0.79 ± 0.03 ^c^	0.34 ± 0.03 ^b^	0.15 ± 0.04 ^ab^	63.80 ± 0.57 ^f^	3.69 ± 0.23 ^d^
4b	25.64 ± 0.22 ^e^	341.84 ± 2.45 ^b^	3.63 ± 0.18 ^a^	1.36 ± 0.16 ^a^	801.26 ± 23.52 ^b^	0.80 ± 0.02 ^c^	0.43 ± 0.04 ^c^	0.16 ± 0.02 ^ab^	66.11 ± 0.44 ^g^	5.43 ± 0.79 ^f^
1c	104.44 ± 0.83 ^kl^	329.25 ± 2.76 ^a^	3.55 ± 0.35 ^a^	1.93 ± 0.32 ^b^	786.04 ± 15.00 ^ab^	0.79 ± 0.05 ^c^	1.31 ± 0.23 ^d^	0.12 ± 0.03 ^a^	55.66 ± 0.94 ^d^	3.00 ± 0.54 ^cd^
2c	98.17 ± 3.43 ^k^	356.80 ± 7.03 ^c^	3.98 ± 0.15 ^ab^	1.76 ± 0.06 ^b^	810.91 ± 16.85 ^b^	0.81 ± 0.03 ^c^	0.49 ± 0.04 ^c^	0.14 ± 0.04 ^a^	68.61 ± 0.60 ^h^	3.53 ± 0.14 ^d^
3c	28.34 ± 0.53 ^f^	373.63 ± 5.72 ^d^	3.82 ± 0.37 ^ab^	2.47 ± 0.31 ^c^	805.03 ± 13.65 ^b^	0.81 ± 0.02 ^c^	0.35 ± 0.05 ^b^	0.13 ± 0.03 ^a^	68.95 ± 0.21 ^h^	3.44 ± 0.22 ^d^
4c	21.26 ± 1.13 ^c^	360.70 ± 4.19 ^c^	3.74 ± 0.08 ^a^	2.37 ± 0.16 ^c^	787.99 ± 14.87 ^ab^	0.79 ± 0.01 ^c^	0.30 ± 0.01 ^b^	0.21 ± 0.07 ^ab^	69.88 ± 0.12 ^i^	4.60 ± 0.50 ^ef^
1d	102.27 ± 1.64 ^k^	332.42 ± 2.13 ^a^	3.97 ± 0.23 ^ab^	1.39 ± 0.08 ^a^	775.21 ± 12.73 ^ab^	0.78 ± 0.01 ^c^	1.36 ± 0.19 ^d^	0.11 ± 0.03 ^a^	57.30 ± 0.62 ^e^	4.26 ± 0.29 ^e^
2d	58.03 ± 0.99 ^h^	337.06 ± 2.74 ^a^	3.43 ± 0.41 ^a^	1.81 ± 0.29 ^b^	796.63 ± 18.44 ^ab^	0.80 ± 0.02 ^c^	0.26 ± 0.04 ^ab^	0.18 ± 0.02 ^b^	68.78 ± 0.82 ^h^	3.17 ± 0.08 ^d^
3d	24.51 ± 1.26 ^d^	349.09 ± 3.66 ^bc^	3.93 ± 0.03 ^a^	1.33 ± 0.19 ^a^	780.96 ± 12.09 ^ab^	0.78 ± 0.01 ^c^	0.33 ± 0.07 ^ab^	0.09 ± 0.05 ^a^	71.41 ± 0.31 ^j^	3.10 ± 0.05 ^d^
4d	17.07 ± 0.88 ^a^	348.25 ± 6.65 ^bc^	3.74 ± 0.38 ^ab^	1.95 ± 0.34 ^b^	790.96 ± 16.98 ^ab^	0.79 ± 0.02 ^c^	0.33 ± 0.02 ^b^	0.16 ± 0.09 ^ab^	72.72 ± 0.90 ^j^	2.95 ± 0.27 ^cd^
1e	105.83 ± 1.11 ^l^	344.39 ± 2.26 ^b^	3.89 ± 0.28 ^ab^	2.24 ± 0.33 ^c^	783.34 ± 11.05 ^ab^	0.78 ± 0.01 ^c^	1.37 ± 0.07 ^d^	0.15 ± 0.05 ^ab^	54.42 ± 0.63 ^d^	2.57 ± 0.22 ^c^
2e	23.35 ± 0.74 ^d^	340.19 ± 2.25 ^b^	3.62 ± 0.42 ^ab^	1.86 ± 0.29 ^b^	780.95 ± 4.34 ^ab^	0.78 ± 0.04 ^c^	0.20 ± 0.02 ^a^	0.12 ± 0.03 ^a^	63.04 ± 0.40 ^f^	2.51 ± 0.11 ^c^
3e	27.86 ± 0.78 ^f^	377.06 ± 1.47 ^d^	3.57 ± 0.47 ^ab^	1.27 ± 0.19 ^a^	797.77 ± 27.17 ^ab^	0.80 ± 0.03 ^c^	0.20 ± 0.02 ^a^	0.08 ± 0.02 ^a^	63.86 ± 0.48 ^f^	2.59 ± 0.22 ^c^
4e	24.03 ± 0.23 ^d^	356.80 ± 5.06 ^c^	3.89 ± 0.16 ^ab^	1.79 ± 0.10 ^b^	780.16 ± 12.73 ^ab^	0.78 ± 0.01 ^c^	0.39 ± 0.03 ^bc^	0.12 ± 0.05 ^ab^	63.68 ± 0.84 ^f^	2.62 ± 0.05 ^c^

Significant differences (*p* < 0.05) in the same column have been marked with different superscript letters (a–m). Me20—Merlot vintage 2020 sample before ageing; a—stainless-steel tank; b—Excellence wooden barrel with medium toasting; c—Excellence wooden barrel with medium-plus toasting; d—Excellence wooden barrel with medium-long toasting; e—Premium wooden barrel with medium toasting; 1a–1e—sampling in June 2021; 2a–2e—sampling in September 2021; 3a–3e—sampling in December 2021; 4a–4e—sampling in March 2022.

**Table 11:** Element content in Merlot vintage 2021 and samples obtained during 12-month ageing in different vessels.

Sample	B (mg/L)	Na (mg/L)	Al (μg/L)	Ca (mg/L)	V (μg/L)	Cr (μg/L)	Mn (μg/L)	Fe (μg/L)	Co (μg/L)	Ni (μg/L)
Me21	2.83 ± 0.02 ^bc^	4.25 ± 0.03 ^d^	138.03 ± 5.73 ^e^	5.71 ± 0.14 ^b^	0.58 ± 0.03 ^c^	2.87 ± 0.04 ^f^	683.90 ± 10.05 ^a^	987.41 ± 10.01 ^h^	4.95 ± 0.11 ^ab^	15.69 ± 0.08 ^h^
1A	2.88 ± 0.02 ^c^	4.24 ± 0.08 ^d^	112.95 ± 8.51 ^bc^	5.89 ± 0.56 ^ab^	0.56 ± 0.01 ^c^	2.92 ± 0.07 ^f^	699.93 ± 19.27 ^ab^	872.39 ± 15.29 ^f^	4.86 ± 0.19 ^ab^	15.17 ± 0.09 ^g^
2A	2.79 ± 0.10 ^b^	3.98 ± 0.03 ^bc^	117.46 ± 6.30 ^c^	5.50 ± 0.12 ^b^	0.47 ± 0.09 ^bc^	2.44 ± 0.03 ^d^	682.16 ± 15.40 ^a^	809.08 ± 19.39 ^e^	4.92 ± 0.16 ^ab^	14.44 ± 0.02 ^d^
3A	2.63 ± 0.06 ^ab^	3.74 ± 0.03 ^a^	125.97 ± 2.82 ^d^	5.05 ± 0.09 ^a^	0.50 ± 0.10 ^bc^	2.24 ± 0.05 ^c^	673.61 ± 16.28 ^a^	751.89 ± 20.00 ^d^	4.64 ± 0.19 ^a^	14.15 ± 0.06 ^c^
4A	2.79 ± 0.05 ^b^	4.23 ± 0.02 ^d^	118.83 ± 2.28 ^c^	4.99 ± 0.33 ^a^	0.35 ± 0.04 ^a^	1.35 ± 0.09 ^b^	672.83 ± 16.32 ^a^	660.07 ± 15.73 ^b^	4.76 ± 0.14 ^a^	13.15 ± 0.07 ^b^
1B	2.73 ± 0.04 ^b^	4.03 ± 0.02 ^c^	112.87 ± 5.72 ^c^	5.52 ± 0.11 ^b^	0.48 ± 0.07 ^b^	2.48 ± 0.05 ^d^	691.27 ± 11.45 ^ab^	805.33 ± 11.25 ^e^	4.92 ± 0.16 ^ab^	14.72 ± 0.04 ^e^
2B	2.75 ± 0.07 ^b^	3.88 ± 0.05 ^b^	110.91 ± 4.53 ^c^	5.60 ± 0.14 ^b^	0.47 ± 0.02 ^b^	2.65 ± 0.02 ^e^	692.72 ± 10.96 ^ab^	836.93 ± 14.08 ^e^	4.91 ± 0.11 ^ab^	15.18 ± 0.13 ^g^
3B	2.59 ± 0.03 ^a^	3.77 ± 0.01 ^a^	102.65 ± 1.72 ^b^	4.96 ± 0.28 ^a^	0.43 ± 0.01 ^b^	2.49 ± 0.03 ^d^	691.29 ± 20.80 ^ab^	715.60 ± 14.25 ^c^	4.65 ± 0.11 ^a^	15.07 ± 0.14 ^g^
4B	2.98 ± 0.01 ^d^	4.14 ± 0.08 ^cd^	99.06 ± 6.14 ^ab^	5.12 ± 0.12 ^a^	0.38 ± 0.03 ^ab^	1.42 ± 0.09 ^b^	708.21 ± 14.76 ^b^	691.83 ± 15.03 ^bc^	5.01 ± 0.11 ^b^	14.63 ± 0.09 ^e^
1C	2.82 ± 0.04 ^bc^	3.94 ± 0.09 ^bc^	124.09 ± 1.49 ^d^	5.55 ± 0.07 ^ab^	0.44 ± 0.02 ^b^	3.42 ± 0.04 ^h^	700.57 ± 21.76 ^b^	848.21 ± 15.15 ^ef^	4.97 ± 0.13 ^ab^	14.92 ± 0.07 ^f^
2C	2.85 ± 0.05 ^bc^	3.96 ± 0.05 ^bc^	119.64 ± 1.84 ^c^	5.71 ± 0.09 ^b^	0.42 ± 0.02 ^b^	2.60 ± 0.01 ^e^	699.88 ± 10.53 ^ab^	870.43 ± 7.44 ^f^	4.92 ± 0.08 ^ab^	15.39 ± 0.08 ^g^
3C	2.66 ± 0.04 ^a^	3.93 ± 0.06 ^bc^	111.05 ± 1.69 ^c^	5.34 ± 0.20 ^ab^	0.45 ± 0.03 ^b^	2.47 ± 0.04 ^d^	687.63 ± 8.63 ^a^	757.24 ± 11.16 ^d^	4.80 ± 0.10 ^a^	14.87 ± 0.06 ^f^
4C	2.89 ± 0.04 ^c^	4.20 ± 0.09 ^cd^	91.26 ± 7.82 ^a^	4.97 ± 0.16 ^a^	0.34 ± 0.03 ^a^	0.90 ± 0.08 ^a^	680.02 ± 18.88 ^a^	647.43 ± 22.13 ^ab^	4.67 ± 0.05 ^a^	12.41 ± 0.08 ^a^
1D	2.82 ± 0.11 ^bc^	4.30 ± 0.04 ^c^	122.48 ± 9.92 ^cd^	5.82 ± 0.33 ^b^	0.49 ± 0.04 ^bc^	2.57 ± 0.10 ^d^	696.06 ± 4.90 ^ab^	863.62 ± 6.51 ^f^	4.77 ± 0.09 ^a^	15.44 ± 0.13 ^g^
2D	2.68 ± 0.05 ^ab^	4.29 ± 0.07 ^c^	118.97 ± 2.24 ^c^	5.68 ± 0.45 ^ab^	0.47 ± 0.03 ^bc^	2.48 ± 0.08 ^d^	698.27 ± 8.20 ^ab^	819.81 ± 10.76 ^e^	4.96 ± 0.07 ^ab^	15.11 ± 0.02 ^g^
3D	2.65 ± 0.07 ^ab^	3.91 ± 0.09 ^bc^	109.11 ± 8.26 ^bc^	5.36 ± 0.42 ^ab^	0.39 ± 0.05 ^ab^	2.17 ± 0.08 ^c^	703.47 ± 17.19 ^b^	723.22 ± 6.19 ^c^	4.95 ± 0.12 ^ab^	14.66 ± 0.08 ^e^
4D	2.99 ± 0.05 ^d^	4.17 ± 0.11 ^cd^	122.07 ± 3.24 ^cd^	5.38 ± 0.12 ^ab^	0.34 ± 0.06 ^ab^	1.35 ± 0.05 ^b^	705.07 ± 7.81 ^b^	666.84 ± 4.05 ^b^	5.13 ± 0.19 ^b^	15.24 ± 0.03 ^g^
1E	2.83 ± 0.11 ^bc^	4.01 ± 0.02 ^c^	134.40 ± 6.97 ^e^	5.82 ± 0.33 ^b^	0.52 ± 0.03 ^c^	2.93 ± 0.02 ^f^	702.99 ± 1.68 ^b^	895.14 ± 5.22 ^g^	4.93 ± 0.04 ^ab^	14.33 ± 0.08 ^c^
2E	2.86 ± 0.06 ^bc^	4.21 ± 0.06 ^d^	129.62 ± 3.04 ^d^	5.89 ± 0.41 ^b^	0.45 ± 0.08 ^ab^	3.06 ± 0.05 ^g^	716.11 ± 20.80 ^bc^	841.88 ± 16.64 ^ef^	4.99 ± 0.19 ^ab^	16.22 ± 0.05 ^i^
3E	2.59 ± 0.02 ^a^	4.12 ± 0.09 ^cd^	115.72 ± 3.29 ^c^	5.51 ± 0.92 ^ab^	0.55 ± 0.05 ^c^	2.96 ± 0.03 ^f^	704.87 ± 15.47 ^b^	713.74 ± 8.58 ^c^	4.82 ± 0.11 ^ab^	14.58 ± 0.08 ^e^
4E	2.85 ± 0.05 ^bc^	4.01 ± 0.11 ^bc^	99.84 ± 1.31 ^ab^	5.31 ± 0.20 ^ab^	0.51 ± 0.04 ^c^	1.54 ± 0.03 ^b^	753.07 ± 14.01 ^c^	613.02 ± 20.20 ^a^	4.86 ± 0.16 ^ab^	12.45 ± 0.04 ^a^
Sample	Cu (μg/L)	Zn (μg/L)	As (μg/L)	Se (μg/L)	Sr (μg/L)	Mo (μg/L)	Sn (μg/L)	Sb (μg/L)	Ba (μg/L)	Pb (μg/L)
Me21	68.01 ± 1.45 ^g^	387.85 ± 3.63 ^d^	3.48 ± 0.03 ^k^	2.27 ± 0.08 ^h^	866.77 ± 14.95 ^f^	0.36 ± 0.04 ^a^	0.14 ± 0.01 ^cd^	0.49 ± 0.03 ^e^	55.53 ± 0.79 ^f^	1.73 ± 0.03 ^e^
1A	59.48 ± 1.35 ^ef^	393.73 ± 9.93 ^d^	3.35 ± 0.03 ^j^	1.89 ± 0.05 ^fg^	875.16 ± 33.58 ^f^	0.36 ± 0.06 ^a^	0.18 ± 0.01 ^d^	0.22 ± 0.02 ^c^	55.24 ± 0.72 ^f^	1.58 ± 0.09 ^cd^
2A	69.57 ± 1.42 ^g^	345.88 ± 4.02 ^b^	3.52 ± 0.01 ^k^	1.54 ± 0.08 ^c^	846.70 ± 11.78 ^f^	0.37 ± 0.02 ^a^	0.16 ± 0.01 ^d^	0.16 ± 0.01 ^b^	51.30 ± 1.03 ^e^	1.49 ± 0.03 ^c^
3A	69.01 ± 1.34 ^g^	331.89 ± 6.59 ^a^	3.05 ± 0.06 ^h^	1.56 ± 0.03 ^c^	720.10 ± 21.01 ^d^	0.32 ± 0.04 ^a^	0.12 ± 0.02 ^c^	0.17 ± 0.02 ^b^	41.27 ± 1.05 ^b^	1.34 ± 0.08 ^ab^
4A	45.11 ± 0.98 ^b^	341.47 ± 8.27 ^ab^	1.02 ± 0.04 ^a^	1.53 ± 0.03 ^c^	591.30 ± 7.99 ^a^	0.75 ± 0.10 ^b^	-	0.66 ± 0.01 ^f^	37.86 ± 0.39 ^a^	1.76 ± 0.07 ^e^
1B	60.72 ± 1.47 ^f^	382.25 ± 11.32 ^cd^	3.28 ± 0.04 ^i^	1.63 ± 0.04 ^de^	820.53 ± 15.55 ^f^	0.38 ± 0.07 ^a^	0.11 ± 0.01 ^c^	0.15 ± 0.03 ^b^	51.43 ± 1.07 ^e^	1.53 ± 0.03 ^cd^
2B	59.05 ± 0.39 ^f^	367.99 ± 10.41 ^c^	3.31 ± 0.08 ^i^	1.69 ± 0.02 ^de^	868.62 ± 20.06 ^f^	0.37 ± 0.03 ^a^	0.08 ± 0.01 ^b^	0.14 ± 0.02 ^b^	58.41 ± 0.57 ^g^	1.57 ± 0.01 ^d^
3B	51.46 ± 1.25 ^d^	368.78 ± 8.83 ^c^	2.90 ± 0.09 ^gh^	1.96 ± 0.02 ^g^	633.17 ± 6.85 ^b^	0.40 ± 0.05 ^a^	0.09 ± 0.02 ^bc^	0.16 ± 0.02 ^b^	40.11 ± 0.29 ^b^	1.22 ± 0.07 ^a^
4B	47.41 ± 0.59 ^c^	358.05 ± 9.22 ^bc^	1.69 ± 0.01 ^d^	1.71 ± 0.04 ^e^	638.52 ± 8.02 ^b^	0.55 ± 0.03 ^b^	-	0.59 ± 0.06 ^e^	45.77 ± 0.93 ^c^	1.92 ± 0.02 ^f^
1C	59.77 ± 1.85 ^ef^	393.26 ± 4.20 ^d^	3.15 ± 0.08 ^hi^	1.47 ± 0.08 ^bc^	812.57 ± 20.17 ^f^	0.37 ± 0.05 ^a^	0.04 ± 0.01 ^a^	0.13 ± 0.02 ^b^	50.84 ± 1.18 ^e^	1.36 ± 0.02 ^b^
2C	52.11 ± 1.15 ^d^	327.00 ± 10.76 ^a^	2.90 ± 0.03 ^g^	1.82 ± 0.02 ^f^	858.20 ± 12.89 ^f^	0.41 ± 0.05 ^a^	0.08 ± 0.0.1 ^b^	0.14 ± 0.01 ^b^	57.76 ± 0.33 ^fg^	1.67 ± 0.08 ^de^
3C	45.32 ± 1.09 ^b^	370.17 ± 6.94 ^c^	3.03 ± 0.04 ^h^	1.55 ± 0.02 ^c^	778.82 ± 12.40 ^e^	0.36 ± 0.04 ^a^	0.11 ± 0.01 ^c^	0.17 ± 0.03 ^b^	48.96 ± 0.19 ^d^	1.38 ± 0.05 ^b^
4C	42.05 ± 1.57 ^a^	352.22 ± 2.48 ^b^	1.43 ± 0.06 ^c^	1.72 ± 0.05 ^e^	671.68 ± 4.16 ^c^	0.43 ± 0.06 ^a^	-	0.55 ± 0.05 ^e^	44.15 ± 0.71 ^c^	1.55 ± 0.03 ^cd^
1D	58.84 ± 1.31 ^e^	394.01 ± 7.68 ^d^	3.61 ± 0.05 ^l^	1.51 ± 0.02 ^c^	847.38 ± 4.80 ^f^	0.38 ± 0.04 ^a^	0.08 ± 0.01 ^b^	0.16 ± 0.02 ^b^	56.54 ± 0.82 ^f^	1.89 ± 0.02 ^f^
2D	53.29 ± 1.64 ^d^	386.15 ± 4.05 ^d^	2.79 ± 0.01 ^fg^	1.57 ± 0.08 ^cd^	821.91 ± 20.74 ^f^	0.37 ± 0.05 ^a^	0.08 ± 0.01 ^b^	0.11 ± 0.03 ^b^	57.19 ± 1.04 ^fg^	1.70 ± 0.06 ^e^
3D	56.97 ± 1.94 ^e^	376.63 ± 1.13 ^c^	2.79 ± 0.05 ^fg^	1.12 ± 0.07 ^a^	738.86 ± 14.98 ^d^	0.39 ± 0.02 ^a^	0.08 ± 0.01 ^b^	0.13 ± 0.01 ^b^	43.42 ± 0.59 ^c^	1.57 ± 0.06 ^cd^
4D	52.16 ± 1.39 ^d^	375.92 ± 11.90 ^cd^	1.25 ± 0.02 ^b^	1.23 ± 0.08 ^ab^	742.47 ± 12.73 ^d^	0.38 ± 0.02 ^a^	-	0.41 ± 0.02 ^d^	48.77 ± 0.78 ^d^	1.73 ± 0.06 ^e^
1E	56.64 ± 1.21 ^e^	402.77 ± 5.85 ^d^	2.72 ± 0.02 ^f^	1.66 ± 0.02 ^de^	846.66 ± 7.37 ^f^	0.37 ± 0.02 ^a^	0.08 ± 0.01 ^b^	0.06 ± 0.01 ^a^	56.04 ± 0.55 ^f^	1.49 ± 0.02 ^c^
2E	53.76 ± 1.04 ^d^	392.62 ± 6.60 ^d^	2.85 ± 0.04 ^g^	1.52 ± 0.03 ^c^	825.17 ± 10.48 ^f^	0.39 ± 0.07 ^a^	0.08 ± 0.01 ^b^	0.06 ± 0.01 ^a^	56.26 ± 0.77 ^f^	1.54 ± 0.02 ^cd^
3E	53.44 ± 1.80 ^d^	371.64 ± 9.15 ^c^	2.27 ± 0.03 ^e^	1.63 ± 0.02 ^d^	718.65 ± 11.94 ^d^	0.39 ± 0.06 ^a^	0.04 ± 0.01 ^a^	0.11 ± 0.03 ^b^	41.54 ± 0.96 ^b^	1.78 ± 0.10 ^ef^
4E	51.56 ± 1.70 ^d^	370.42 ± 9.09 ^c^	1.19 ± 0.05 ^b^	1.35 ± 0.04 ^b^	726.65 ± 8.41 ^d^	0.39 ± 0.05 ^a^	-	0.40 ± 0.03 ^d^	44.03 ± 0.74 ^c^	1.64 ± 0.06 ^de^

Significant differences (*p* < 0.05) in the same column have been marked with different superscript letters (a–l). Me21—Merlot vintage 2021 sample before ageing; A—stainless-steel tank; B—Excellence wooden barrel with medium toasting; C—Excellence wooden barrel with medium-plus toasting; D—Excellence wooden barrel with medium-long toasting; E—Premium wooden barrel with medium toasting; 1A–1E—sampling in August 2022; 2A–2E—sampling in November 2022; 3A–3E—sampling in February 2023; 4A–4E—sampling in May 2023.

The change of concentrations of elements was observed in all samples, and there were some similarities between the two wine vintages. The initial concentrations of Al, V, Fe, Ni, Cu, Zn, As, and Se were as follows: 146.89 μg/L, 0.43 μg/L, 874.73 μg/L, 14.92 μg/L, 186.26 μg/L, 394.56 μg/L, 5.87 μg/L, and 4.46 μg/L in Me20; 138.03 μg/L, 0.58 μg/L, 987.41 μg/L, 15.69 μg/L, 68.01 μg/L, 387.85 μg/L, 3.48 μg/L, and 2.27 μg/L in Me21. After 12 months of the ageing of both wine vintages, the concentrations of the above-mentioned elements decreased in all vessels.

The highest concentrations of all elements were measured for B, Na, and Ca (3.50, 4.06, and 5.62 mg/L in the initial Me20; 2.83, 4.25, and 5.71 mg/L in the initial Me21, respectively). The initial concentration of Na in the Me20 wine (4.06 mg/L) was significantly decreased after 12 months of ageing in SST (3.67 mg/L). Further, in the rest of the vessels, the concentration of Na after 12 months was similar to the initial one. Further, the concentration of Na in Me21 samples did not significantly differ from the initial one after 12 months of ageing in any vessel, except in the PMT barrel where it was lower (4.01 mg/L). After 12 months of ageing, the concentration of Ca among Me20 samples was the lowest in the SST, and a slight increase was observed in EMT, EMT+, and EMLT barrels. In all vessels, the concentration of Ca in the Me21 samples decreased after 12 months of ageing, and the lowest was measured in SST (4.99 mg/L). The concentration of B did not significantly change in most vessels in both wines, compared to the initial concentration. The exceptions were EMT and EMLT barrels for Me21, where the highest concentration of B was measured. On the other hand, in these barrels, along with EMT+, the lowest concentration of B was measured for the 2020 vintage Merlot.

In both Merlot wines, the concentration of manganese (Mn) did not show significant changes during ageing, except in the PMT barrel, where a 10% increase was observed in both vintages. The concentration of chromium (Cr) decreased during ageing in both wines, except for the Me20 wine aged in the EMT+ barrel, where no significant change was noted (concentration remained at 5.11 μg/L). In contrast, the Me21 wine from the EMT+ barrel had the lowest Cr concentration after 12 months of ageing (0.90 μg/L). The concentration of cobalt (Co) in Me20 samples from SST, EMT, and PMT barrels showed no significant difference compared to the initial concentration (3.13 μg/L), although a slight decrease was observed in the EMT+ and EMLT barrels. In the Me21 samples, the lowest Co concentrations were found in the SST and EMT+ barrels after 12 months of ageing (4.76 and 4.67 μg/L, respectively), while the highest concentrations were observed in the EMT and EMLT barrels (5.01 and 5.13 μg/L, respectively). Molybdenum (Mo) concentrations increased in all vessels after 12 months of ageing in Me20, but in Me21, this increase was limited to the SST and EMT barrels. The SST barrel also favoured an increase in antimony (Sb), with the highest concentrations observed after 12 months in both Me20 and Me21 (0.26 μg/L and 0.66 μg/L, respectively).

Strontium (Sr) concentrations showed a notable decrease in most vessels for both vintages. In Me20, the lowest Sr concentration was observed in the SST barrel (745.44 μg/L), while the highest was measured in the EMT barrel (801.26 μg/L). In Me21, the SST barrel yielded the lowest Sr concentration (591.30 μg/L), with a decrease observed in other vessels compared to the initial concentration (866.77 μg/L). The initial concentration of tin (Sn) in Me20 was 1.63 mg/L, with no significant change after 3 months of storage. However, after 12 months of ageing, Sn concentrations significantly decreased to between 0.30 and 0.43 μg/L. In the initial Me21 wine, the concentration of Sn was 0.14 μg/L, which varied during ageing, but it was undetectable in any of the samples after 12 months. Barium (Ba) concentrations increased in all Me20 wine samples after 12 months of ageing, particularly in the EMLT barrel (72.72 μg/L). Conversely, Ba concentrations decreased in all Me21 samples after ageing, with the most significant decrease observed in the SST barrel (37.86 μg/L). Lead (Pb) concentrations in Me20 initially were 2.02 μg/L. After 12 months of ageing, Pb concentrations decreased to 1.60 μg/L in the SST barrel but increased in all other vessels, especially in the EMT barrel (5.43 μg/L). In Me21, the highest Pb concentration after ageing was observed in the EMT barrel (1.92 μg/L), while the lowest was measured in the EMT+ barrel.

The initial content of elements in the 2020 and 2021 vintage Merlot differed; therefore, different behaviours of those elements were observed during ageing in different vessels, comparing two wine vintages. For easier comparison and better insight into the element profile changes of both wines, principal component analysis (PCA) was performed and presented in Figure 2.

At the PCA biplot, it can be observed that Principal Component 1 (PC1) with 69.22% of total variance separates the samples on the 2020 vintage Merlot (positive side) and 2021 vintage Merlot (negative side). This indicates that the element profile of the two vintages of Merlot red wine significantly differed. The initial wines, Me20 and Me21, were closer to the corresponding samples obtained from the SST and wooden barrels in the early stage of ageing, especially after 3 and 6 months of ageing. A longer ageing time resulted in noticeable changes in element content, especially in wooden barrels. The Me20 samples after 9 and 12 months of ageing were mostly clustered at the positive side of PC1 and PC2, around elements Na, Cr, As, Al, Ca, Pb, and Sn. Samples obtained after shorter ageing (3 and 6 months) were clustered around Sn, Mo, B, and Cu at the positive side of PC1 and the negative side of PC2. The shorter ageing time of the Me21 wine in different barrels placed those samples in the first quadrant, and they were correlated with Mn, Co, V, Ni, Zn, and Fe. After 9 and 12 months of ageing, changes occurred, placing the samples of Me21 in the lower quadrant (negative sides of PC1 and PC2). However, it could be observed that, besides vintage, the ageing time had a higher influence than the vessel type.

Table 12 and Table 13 present the summative data of analysis of variance (ANOVA) of all results obtained for analysed samples: source of variation (SV), degrees of freedom (df), sum of squares (SS), mean squares (MS), F and F_critical_ value, *p*-value.

**Table 12:** ANOVA summative table for Merlot 2020 results.

Characteristic	SV	df	SS	MS	F	*p*-Value	F_critical_
Ethanol	ANOVA—Between groups	20	5.696	0.285	23.924	<0.0001	2.080
Total sugar		20	0.596	0.03	5.962	0.000	2.080
pH		20	7.631	0.382	0.97	0.526	2.080
Total acidity		20	0.821	0.041	8.214	<0.0001	2.080
Volatile acidity		20	0.094	0.005	23.562	<0.0001	2.080
Malic acid		20	0.28	0.014	2.8	0.012	2.080
Lactic acid		20	0.473	0.024	4.729	0.000	2.080
Density		20	0	0	1.011	0.489	2.080
TPC		20	0.267	0.014	16.744	<0.0001	2.080
TFC		20	0.202	0.010	11.139	<0.0001	2.080
MAC		20	1429.095	71.455	115.379	<0.0001	2.080
PC		20	2798.028	139.901	331.730	<0.0001	2.080
CTC		20	76,207.863	3810.393	108.477	<0.0001	2.080
HTC		20	3571.053	238.070	13.206	<0.0001	2.080
DPPH		20	48.435	2.422	25.546	<0.0001	2.080
ABTS		20	158.707	7.935	52.731	<0.0001	2.080
FRAP		20	716.328	35.816	106.778	<0.0001	2.080
CUPRAC		20	13,274.190	663.710	452.779	<0.0001	2.080
Quercetin		20	100.149	5.007	724.219	<0.0001	2.080
Hyperoside		20	12.786	0.639	112.438	<0.0001	2.080
Gallic acid		20	274.561	13.728	108.813	<0.0001	2.080
p-coumaric acid		20	50.189	2.509	161.650	<0.0001	2.080
Caftaric acid		20	3593.072	179.654	784.611	<0.0001	2.080
Coutaric acid		20	1152.163	57.608	409.010	<0.0001	2.080
Caffeic acid		20	2030.859	101.543	1309.186	<0.0001	2.080
(+)-catechin		20	469.009	23.450	179.993	<0.0001	2.080
(−)-epicatechin		20	148.103	7.405	35.931	<0.0001	2.080
malvidin-3-glucoside		20	70.598	3.530	195.486	<0.0001	2.080
delphinidin-3-glucoside		20	4.001	0.200	74.216	<0.0001	2.080
L*		20	2.912	0.146	238.872	<0.0001	2.080
a*		20	1.275	0.064	104.559	<0.0001	2.080
b*		20	0.264	0.013	19.548	<0.0001	2.080
°h		20	3582.646	179.132	415.262	<0.0001	2.080
C*		20	0.937	0.047	98.342	<0.0001	2.080
ΔE*		20	0.776	0.041	204.116	<0.0001	2.080
B		20	0.384	0.019	0.981	0.515	2.080
Na		20	2.132	0.107	4.501	0.001	2.080
Al		20	3472.021	173.601	1.867	0.082	2.080
Ca		20	13.577	0.679	7.071	<0.0001	2.080
V		20	0.224	0.011	4.412	0.001	2.080
Cr		20	99.260	4.963	12.855	<0.0001	2.080
Mn		20	20,985.595	1049.280	2.598	0.018	2.080
Fe		20	206,761.994	10,338.050	12.764	<0.0001	2.080
Co		20	0.465	0.023	2.594	0.018	2.080
Ni		20	74.639	3.732	29.226	<0.0001	2.080
Cu		20	132,487.497	6624.375	1678.773	<0.0001	2.080
Zn		20	13,117.039	655.852	18.662	<0.0001	2.080
As		20	15.766	0.788	3.244	0.005	2.080
Se		20	52.866	2.643	12.505	<0.0001	2.080
Sr		20	18,897.417	944.871	1.844	0.086	2.080
Mo		20	0.494	0.025	9.566	0.001	2.080
Sn		20	10.796	0.540	21.133	<0.0001	2.080
Sb		20	0.549	0.027	4.601	<0.0001	2.080
Ba		20	4720.815	236.041	309.765	<0.0001	2.080
Pb		20	39.768	1.988	9.225	<0.0001	2.080

SV—the source of variation; df—degree of freedom; SS—sum of squares; MS—mean of squares.

**Table 13:** ANOVA summative table for Merlot 2021 results.

Characteristic	SV	df	SS	MS	F	*p*-Value	F_critical_
Ethanol	ANOVA—Between groups	20	78.265	3.913	1.148	0.377	2.080
Total sugar		20	0.609	0.030	6.09	<0.0001	2.080
pH		20	0.012	0.001	0.598	0.872	2.080
Total acidity		20	1.805	0.090	18.048	<0.0001	2.080
Volatile acidity		20	0.045	0.002	11.162	<0.0001	2.080
Malic acid		20	0.509	0.025	5.09	0.000	2.080
Lactic acid		20	0.719	0.036	7.190	<0.0001	2.080
Density		20	0.000	0.000	0.472	0.951	2.080
TPC		20	0.221	0.011	16.341	<0.0001	2.080
TFC		20	0.142	0.007	19.597	<0.0001	2.080
MAC		20	3346.908	167.345	153.060	<0.0001	2.080
PC		20	287.099	14.355	22.273	<0.0001	2.080
CTC		20	18,227.359	911.368	36.316	<0.0001	2.080
HTC		20	23,765.762	1584.384	38.542	<0.0001	2.080
DPPH		20	147.515	7.376	37.266	<0.0001	2.080
ABTS		20	470.189	23.509	87.887	<0.0001	2.080
FRAP		20	488.193	24.410	103.019	<0.0001	2.080
CUPRAC		20	43,736.899	2186.845	3859.722	<0.0001	2.080
Quercetin		20	21.689	1.084	281.844	<0.0001	2.080
Hyperoside		20	51.570	2.579	392.951	<0.0001	2.080
Gallic acid		20	233.911	11.696	332.620	<0.0001	2.080
p-coumaric acid		20	45.970	2.298	946.438	<0.0001	2.080
Caftaric acid		20	2640.296	132.015	1129.135	<0.0001	2.080
Coutaric acid		20	198.648	9.932	295.607	<0.0001	2.080
Caffeic acid		20	854.704	42.735	1776.404	<0.0001	2.080
(+)-catechin		20	813.123	40.656	158.695	<0.0001	2.080
(−)-epicatechin		20	417.995	20.900	335.649	<0.0001	2.080
malvidin-3-glucoside		20	46.682	2.334	107.776	<0.0001	2.080
delphinidin-3-glucoside		20	8.508	0.425	50.587	<0.0001	2.080
L*		20	1.493	0.075	118.765	<0.0001	2.080
a*		20	2.875	0.144	359.314	<0.0001	2.080
b*		20	0.397	0.020	69.403	<0.0001	2.080
°h		20	7313.641	365.682	420.398	<0.0001	2.080
C*		20	1.523	0.076	159.936	<0.0001	2.080
ΔE*		20	1.060	0.056	279.063	<0.0001	2.080
B		20	0.540	0.027	3.920	0.002	2.080
Na		20	1.134	0.057	6.464	<0.0001	2.080
Al		20	5481.389	274.069	5.012	0	2.080
Ca		20	3.772	0.189	0.849	0.642	2.080
V		20	0.194	0.010	2.051	0.055	2.080
Cr		20	17.795	0.890	134.230	<0.0001	2.080
Mn		20	11,704.654	585.233	1.373	0.238	2.080
Fe		20	376,520.553	18,826.028	49.284	<0.0001	2.080
Co		20	0.631	0.032	0.905	0.587	2.080
Ni		20	49.000	2.450	194.297	<0.0001	2.080
Cu		20	2300.212	115.011	30.118	<0.0001	2.080
Zn		20	18,437.452	921.873	7.507	<0.0001	2.080
As		20	27.519	1.376	309.365	<0.0001	2.080
Se		20	2.441	0.122	24.551	<0.0001	2.080
Sr		20	303,793.702	15,189.685	33.850	<0.0001	2.080
Mo		20	0.326	0.016	3.234	0.005	2.080
Sn		20	3.365	0.224	997.122	<0.0001	2.080
Sb		20	1.383	0.069	48.730	<0.0001	2.080
Ba		20	1774.898	88.745	71.715	<0.0001	2.080
Pb		20	1.298	0.065	10.765	<0.0001	2.080

SV—the source of variation; df—degree of freedom; SS—sum of squares; MS—mean of squares.

Red wines are often subjected to maturation and ageing. The most common vessels for this vinification stage are stainless-steel tanks or wooden barrels. In this study, two vintages (2020 and 2021) of Merlot red wine have aged for 12 months in five different vessels: one stainless-steel tank and four oak barrels with different toasting methods. The aim was to determine the stability or change of phenolic compound content, antioxidant activity, colour, and chemical and element composition of the mentioned wines during 12-month ageing and to establish the similarities or differences between two vintages and different vessel types.

From the results of this study, it was observed that the same vinification techniques resulted in similar initial chemical composition, regarding main parameters like ethanol, total sugar, acidity, and even colour. However, differences between the two vintages were more pronounced in phenolic content and antioxidant activity, and in the concentrations of some elements, indicating that conditions in the vineyard influenced the Merlot grape variety.

In this study, a slight decrease in malic acid was followed by a slight lactic acid increase, which is a usual consequence of malolactic fermentation in red wines [16]. The results from a previous study [29] showed that the ageing time affected the wine phenolic content in different wine varieties. They also stated that aged wines had a lower content of monomeric anthocyanins and malvidin-3-glucoside, which is consistent with the results obtained in this study. Anthocyanins in wine are often susceptible to degradation, especially if wine is stored at inadequate temperatures [30]. However, they also tend to react with other compounds, forming new and more stable polymers [31,32]. This is usually accompanied by an increase in polymeric colour percentage [33], as it was also obtained in this study, regarding MAC and PC values in both wines during ageing in different vessels.

The changes of TPC and TFC varied between samples, but, in general, an increase was observed, especially during ageing in the EMT+ barrel. The antioxidant activity was measured with four different assays, two decolourisation assays (DPPH and ABTS) and two assays characterised by an increase in absorbance in the presence of antioxidants (FRAP and CUPRAC). All assays, however, differ in principles and reactions with different compounds, and usually one is not enough to present the whole picture of the antioxidant capacity of the wine [34]. This could be the reason why the changes in antioxidant activity in all samples did not always follow the same trend. Similar observations were obtained in a previous study of the ageing of different red wine varieties in oak barrels over several years [6]. The changes observed in phenolic compounds could be a consequence of their transformation into condensed forms that do not react with reagents used in assays for their determination. Further, the enzymes that are present in wine, especially from the residual yeasts, could lead to changes in the chemical properties of phenolic compounds [35].

During the ageing of Merlot wines used in this study, significant changes in condensed and hydrolysable tannins were observed. Condensed tannins, also known as proanthocyanidins, usually derive from grapes, and they contribute to the wine taste and stability. However, in young wines, especially red ones, higher concentrations of condensed tannins can result in very astringent and bitter wine [36]. The wine ageing process can result in their polymerisation, reaction with anthocyanins, or participating in oxidation reaction. These reactions could lead to colour stabilisation, maturation, reduction in bitterness and astringency, and an increase in the complexity and quality of red wine. This is more pronounced during wine ageing in wooden barrels due to micro-oxygenation, rather than in stainless-steel tanks [37]. This could also result in a decreased concentration of condensed tannins, which was obtained and proved in this study. On the other hand, hydrolysable tannins, like gallotannins, are mostly extracted from wooden barrels, especially in the initial stages of the wine/wood contact and from new or freshly toasted barrels. This type of tannin is naturally occurring in wood, including oak. Therefore, their concentrations could increase during wine ageing in wooden barrels, but, over time, they react with phenolic compounds, increasing wine complexity, structure, and even astringency [38]. In this study, it was observed that only during ageing in wooden barrels, the concentration of hydrolysable tannins increased, while in the initial wine and the SST, they were not detected.

In this study, HPLC analysis was used to identify individual phenolic compounds that are mainly present in red wines. Their concentrations also varied during ageing, which could be a result of different reaction pathways. Wang et al. [39] also studied the effect of different vessels on phenolic compounds content in red wine, and their results showed that gallic acid concentration increases in both stainless-steel tanks and wooden barrels. The increase in gallic acid in wooden barrels is usually a result of the hydrolysis of gallotannins due to lower pH, storage conditions, oxygen presence, etc. [20]. However, as a strong antioxidant, gallic acid undergoes an oxidation process that results in changes in its concentration in wine [40]. Further, the reduction in flavonol concentration during ageing in wooden barrels could be a result of their reaction with sugars, forming flavonol glycosides. On the other hand, an opposite reaction is also possible: the hydrolysis of flavonol glycosides that results in an increase in flavonol concentrations and its variations during ageing [6,41], as was the case in Merlot samples from this study, especially aged in a stainless-steel tank. A different behaviour of flavan-3-ols in the analysed samples was also observed. While the increase in those compounds is usually correlated to their formation from galloylated precursors, their decrease can be explained by their tendency to participate in oxidation and polymerisation reactions with tannins and anthocyanins [42].

Pilet et al. [43] studied the influence of wooden barrels from three different cooperages and with two toasting levels (medium and medium plus) on the phenolic and mineral composition of red wine during 4 and 6 months of ageing. They stated that the change in chemical composition was not statistically significant in all samples. A similar result was obtained in this study, regarding the main chemical composition, while only slight changes or no significant changes were observed for most components. However, the changes in phenolic content could be a result of many factors, like mutual reactions of phenolic compounds, their reactions with other wine compounds, like volatile compounds [44], compounds extracted from wood [45], or a result of the micro-oxygenation phenomena that occurred during ageing in wooden barrels due to wood porosity [46]. Further, changes in element composition also depended on several factors during ageing, but most of the concentration changes are very small or with no significant difference, as stated by Pilet et al. [43].

The concentration of elements in the initial wine and in wines after ageing in different vessels depends on various factors, starting from vineyard conditions (soil composition and vine treatments), vinification techniques (materials used, vessels, and contact with other equipment), and mutual interactions of wine components, which led to different trends of their change [47]. Further, it has been shown that oxygen presence had a great influence on many elements, usually reducing their concentrations, like Cu, Cr, Se, Fe, Al, or Zn [48]. A previous study showed that Se could act as an antioxidant and browning inhibitor, participating in scavenging reactive oxygen ions [49]. Another previous study [50] showed that manganese was a strong oxidant, but its activity depended on the concentration of Cu and Fe in wine. However, Mn ions can react with oxygen itself, but when higher concentrations of Fe are present, they tend to accelerate the oxidation of Fe, which causes further oxidation reactions. The content of Cu could change due to its reaction with sulphur anion from sulphur oxide and the formation of copper sulphite. The reaction depends on the availability of the substrates and the redox potential of the wine [43,51]. This could explain the decrease in Cu concentrations during the ageing of 2020 and 2021 Merlot in different vessels analysed in this study. Further, the mineral composition of oak wood usually contains many elements, especially Na, Mg, K, and Ca, and the transfer of those elements into wine could be expected, especially when a wooden barrel is toasted [43,52]. Kaya et al. [51] suggested that wood ageing did not significantly affect Sr concentration, measured through the isotopic ratio ^87^Sr/^86^Sr during 30, 60, and 90 days of ageing. In our study, ^88^Sr isotope was determined, and the results showed that its concentration decreased during the ageing of both wine vintages in the SST, but no significant difference was observed during the ageing of Me20 in wooden barrels. However, a slight decrease in Sr content was observed during the longer ageing (9 and 12 months) of Me21. Molybdenum is naturally present in grapes and wine and acts as a co-factor for some enzymes (nitrate reductase and sulphite oxidase), playing an important role in grapevine function. Its content could change due to enzyme activity. However, Mo is also added to the stainless-steel materials for acid corrosion prevention [53]; therefore, it can be transferred in wine during the contact with such materials (like stainless-steel tanks or pipes).

Regarding the CIELab parameters determined in this study, all of the mentioned changes in wine colour pigments and chemical composition only slightly affected the total colour change of Merlot red wines during ageing in different vessels. Slight changes have been observed in most samples in lightness, hue angle, saturation, or even in a decrease in the content of red and blue colour (a* and b* parameters, respectively) after 12 months of ageing in all vessels. However, those changes were not significant when the total colour change (ΔE*) was calculated because it was lower than 1 in all samples. The colour change is not visible to the human eye if ΔE* is lower than 1, or even lower than 5 if the wine is seen through a glass [54].

## 4. Conclusions

This study investigated the differences in the chemical composition, phenolic content, antioxidant activity, and colour between 2020 and 2021 Merlot wines aged in different vessels. The goal was to determine whether the same vinification procedures and ageing conditions would affect both vintages similarly.

There are several key findings. Regarding the main chemical composition and visible colour of the wines, negligible changes were observed in the analysed samples. A consistent decrease in monomeric anthocyanins and an increase in polymeric colour were noted during ageing. The condensed tannins content decreased, while hydrolysable tannins content increased over time. Phenolic composition underwent more pronounced changes during extended ageing, especially in wooden barrels. The changes in the antioxidant activity were closely linked to variations in the phenolic profile. All changes depended on the ageing vessel (stainless-steel tank or oak barrel with different variations of medium toasting and wood grain density), the initial composition of Merlot wines, and interactions among the wine’s components. While this study provides insights into the effects of ageing conditions on Merlot red wine, further research is needed to better understand the influence of these variables on the chemical and phenolic composition of red wine.

## Figures and Tables

**Table 1:** Agilent ICP-MS 7900 operating conditions.

Sample Introduction	PeriPump
Nebuliser Type	MicroMist
Ion Lense Model	x—Lens
RF Power	1550 W
RF Matching	1.70 V
Sample introduction	0.99 L/min
Carrier Gas	15.00 L/min
Plasma Gas	0.90 L/min
Aux Gas	0.02 mL/min
He Gas	27.24 MHz

## Data Availability

The original contributions presented in this study are included in the article. Further inquiries can be directed to the corresponding author.
